# Qualitative analysis and phase of chaos control of the predator-prey model with Holling type-III

**DOI:** 10.1038/s41598-022-23074-3

**Published:** 2022-11-22

**Authors:** Mohammed O. AL-Kaff, Hamdy A. El-Metwally, El-Metwally M. Elabbasy

**Affiliations:** 1 Department of Mathematics, Faculty of Science, Mansoura University, Mansoura, 35516 Egypt; 2 Department of Mathematics, Faculty of Education, Seiyun University, Hadhramout, Yemen

**Keywords:** Animal behaviour, Applied mathematics, Computational science, Pure mathematics

## Abstract

In this study, we investigate the dynamics of a discrete-time with predator-prey system with a Holling-III type functional response model. The center manifold theorem and bifurcation theory are used to create existence conditions for flip bifurcations and Neimark-Sacker bifurcations. Bifurcation diagrams, maximum Lyapunov exponents, and phase portraits are examples of numerical simulations that not only show the soundness of theoretical analysis but also show complicated dynamical behaviors and biological processes. From the point of view of biology, this implies that the tiny integral step size can steady the system into locally stable coexistence. Yet, the large integral step size may lead to instability in the system, producing more intricate and richer dynamics. This also means that when the intrinsic death rate of the predator is high, this leads to a chaotic growth rate of the prey. The model has bifurcation features that are similar to those seen in logistic models. In addition, there is a bidirectional Neimark-Sacker bifurcation for both prey and predator, and therefore we obtain a direct correlation in symbiosis. This means that the higher the growth rate of the prey, the greater the growth rate of the predator. Therefore, the operation of predation has increased. The opposite is also true. Finally, the OGY approach is used to control chaos in the predator and prey model. which led to a new concept which we call bifurcation phase of control chaos.

## Introduction

It is generally recognised that when there are non-overlapping generations in populations, discrete-time models defined by difference equations are more useful and trustworthy than continuous-time models. Furthermore, as compared to continuous models, these models give efficient computing results for numerical simulations as well as richer dynamical properties^1–7^. Many fascinating works on the stability, bifurcation and chaotic occurrences in discrete temporal models have appeared in the literature in recent years^8–15^. Because of its widespread occurrence and relevance, research into the dynamic connection between prey-predator has been and will continue to be a hot issue for a long time. In 1959, a Canadian researcher called Holling^16^ presented the matching functional response function for different sorts of species to show the predation rate of predator population to prey population based on his experimental data, which included three primary categories. Holling types I, II, and III, with Holling-III being the functional response function, i.e., $\dfrac{\alpha x_{n}^{2}}{\beta +x_{n}^{2}}$ is applicable to both terrestrial and marine organisms (applies to whales, deer, and other vertebrates). Since then, research into the functional response of Holling type III has grown in importance as a new avenue for studying predator-prey interactions^17,18^. We consider the following set of equations to describe the dynamics of a prey-predator system:

1 $$\begin{aligned} \left\{ \begin{array}{c} x^{\prime }=ax-\dfrac{bx^{2}y}{e+x^{2}}, \\ y^{\prime }=\dfrac{dbx^{2}y}{e+x^{2}}-cy, \end{array} \right. \end{aligned}$$

where *x* and *y* denote prey population and predator population densities, respectively; *a*, *b*, *c*, *d* and *e* are positive constants, *a* a stands for prey intrinsic growth rate, *b* is the predation coefficient of the predator, which reflects the size of the predator’s ability, *c* is death rate of the predator, *d* is the conversion factor denoting the number of newly born predators for each captured prey and *e* is half capturing saturation and the predation rate.

The term $\left( \frac{bx^{2}}{e+x^{2}}\right)$ denotes the responses function of the predator. This function is termed as holling-III responses function.

Using Euler technique on System (1), we get the following system:

2 $$\begin{aligned} \left\{ \begin{array}{c} x_{n+1}=(1+ah)x_{n}-\dfrac{hbx_{n}^{2}y_{n}}{e+x_{n}^{2}}, \\ y_{n+1}=(1-ch)y_{n}+\dfrac{hdbx_{n}^{2}y_{n}}{e+x_{n}^{2}}, \end{array} \right. \end{aligned}$$

where h is the step size integral, we may assume that the prey is located in a spot isolated from predators where the impact of the death of the newly born prey due to predators can be neglected, including its death due to natural conditions. We are only looking at the capabilities of predators for predation. Thus, in System (2), the predator-prey model with the response function of the third type is given by the following system:

3 $$\begin{aligned} \left\{ \begin{array}{c} x_{n+1}=(1+ah)x_{n}-\dfrac{hbx_{n}^{2}y_{n}}{e+x_{n}^{2}}, \\ y_{n+1}=(1-ch)y_{n}+\dfrac{hbx_{n}^{2}y_{n}}{e+x_{n}^{2}}. \end{array} \right. \end{aligned}$$

In this work, we focus on the dynamical behavior of System (3) in the interior first quadrant $\mathbb {R}_{+}^{2}$ from the standpoint of biology. Specifically, the stability of System the fixed points is discussed. Using the center manifold theorem and bifurcation theory, we strictly establish that System (3) undergoes the flip bifurcation, FB in short, and hopf bifurcation, HB in short. Moreover, the research shows a new phase of chaos control using the feedback control approach to stabilise chaos on unstable paths. Numerical simulations that support our findings.

## Existence and stability of the fixed points

In this section, we present some results related to the existence and stability of the fixed points in the model (3). In System (3) *x* and *y* have to be positive values in order to be biologically viable. We have at most two fixed points under various conditions:

(i) The demise state of the total population $p_{0}(0,0)$,
(ii) The cohabitation state of the prey and predator $p_{1}( \frac{ce}{\sqrt{(b-c)ce}},\frac{ae}{\sqrt{(b-c)ce}})$ is inside fixed point exist for $b>c$.

We rewrite System (3) as follows:

4 $$\begin{aligned} \left\{ \begin{array}{c} x_{n+1}=\eta (x_{n},y_{n})=(1+ah)x_{n}-\dfrac{hbx_{n}^{2}y_{n}}{e+x_{n}^{2}},\\ y_{n+1}=\mu (x_{n}, y_{n})=(1-ch)y_{n}+\dfrac{hbx_{n}^{2}y_{n}}{e+x_{n}^{2}}. \end{array} \right. \end{aligned}$$

The Jacobian matrix (J) of System (4) about the fixed point *p*(*x*, *y*) is given by

5 $$\begin{aligned} J(x,y)=\left( \begin{array}{cc} j_{11} &{} j_{12}\\ j_{21} &{} j_{22} \end{array} \right) , \end{aligned}$$

where

$$\begin{aligned} \begin{array}{ll} j_{11}=\frac{\partial \eta (x_{n},y_{n})}{\partial x_{n}}|_{(x_{n},y_{n})}=1+ah-\frac{2behxy}{(e+x^{2})^2}, \ j_{12}=\frac{\partial \eta (x_{n},y_{n})}{\partial y_{n}}|_{(x_{n},y_{n})}=- \frac{hbx^{2}}{e+x^{2}}, \\ j_{21}=\frac{\partial \mu (x_{n},y_{n})}{\partial x_{n}}|_{(x_{n},y_{n})}=\frac{2behxy}{(e+x^{2})^2}\text { and } j_{22}=\frac{\partial \mu (x_{n},y_{n})}{\partial y_{n}}|_{(x_{n},y_{n})}=1-ch+ \frac{hbx^{2}}{e+x^{2}}. \end{array} \end{aligned}$$

The characteristic equation of the variational matrix can be written as

6 $$\begin{aligned} R^{2}+T(x,y)R+D(x,y)=0, \end{aligned}$$

where this is a one-variable quadratic equation with $T(x,y)=-(j_{11}+j_{22})$ and $D(x,y)=j_{11}j_{22}-j_{12}j_{21}$.

### Lemma 1

^19,20^. Let $\mathcal {F}(R)=R^{2}+TR+D$. Suppose that $\mathcal {F}(1)>0,$ $R_{1}$ and $R_{2}$ are two root of $\mathcal {F}(R)=0$. Then

(i) $\left| R_{1}\right| <1$ and $\left| R_{2}\right| <1$ if and only if $\mathcal {F}(-1)>0$ and $D<1$.
(ii) $\left| R_{1}\right| <1$ and $\left| R_{2}\right| >1$ (or $\left| R_{1}\right| >1$ and $\left| R_{2}\right| <1$) if and only if $\mathcal {F}(-1)<0$.
(iii) $\left| R_{1}\right| >1$ and $\left| R_{2}\right| >1$ if and only if $\mathcal {F}(-1)>0$ and $D>1$.
(iv) $R_{1}=-1$ and $\left| R_{2}\right| \ne 1$ if and only if $\mathcal {F}(-1)=0$ and $T\ne 0,2$.
(v) $R_{1}$ and $R_{2}$ are complex and $\left| R_{1}\right| =\left| R_{2}\right| =1$ if and only if $T^{2}-4D<0$ and $D=1$ .

Let $R_{1}$ and $R_{2}$ be two roots of (6). We recall some definitions of topological types for a fixed point *p*(*x*, *y*). *p*(*x*, *y*) is called a sink if $\left| R_{1}\right| <1$ and $\left| R_{2}\right| <1$. A sink is locally asymptotic stable. *p*(*x*, *y*) is called a source if $\left| R_{1}\right| >1$ and $\left| R_{2}\right| >1$. A source is locally unstable. *p*(*x*, *y*) is called a saddle if $\left| R_{1}\right| <1$ and $\left| R_{2}\right| >1$ (or $\left| R_{1}\right| >1$ and $\left| R_{2}\right| <1$). And *p*(*x*, *y*) is called non-hyperbolic if either $\left| R_{1}\right| =1$ or $\left| R_{2}\right| =1$.

### Theorem 1

For the trivial fixed point $P_{0}(0,0)$, the following statements hold:

1. When $0<h<\frac{2}{c}$ is a saddle point.
2. When $h=\frac{2}{c}$ is a non-hyperbolic fixed point.
3. When $h>\frac{2}{c}$ is a source fixed point.

### Proof

The Jacobian matrix at $p_{0}(0,0)$ takes the following form:

7 $$\begin{aligned} J(p_{0})=\left( \begin{array}{cc} ah+1 &{} 0 \\ 0 &{} 1-ch \end{array} \right) , \end{aligned}$$

which has two eigenvalues $R_{1}$ $=ah+1$ and $R_{2}$ $=1-ch$. Clearly, by applying Lemma 1, we get the result directly.

**Figure 1:**
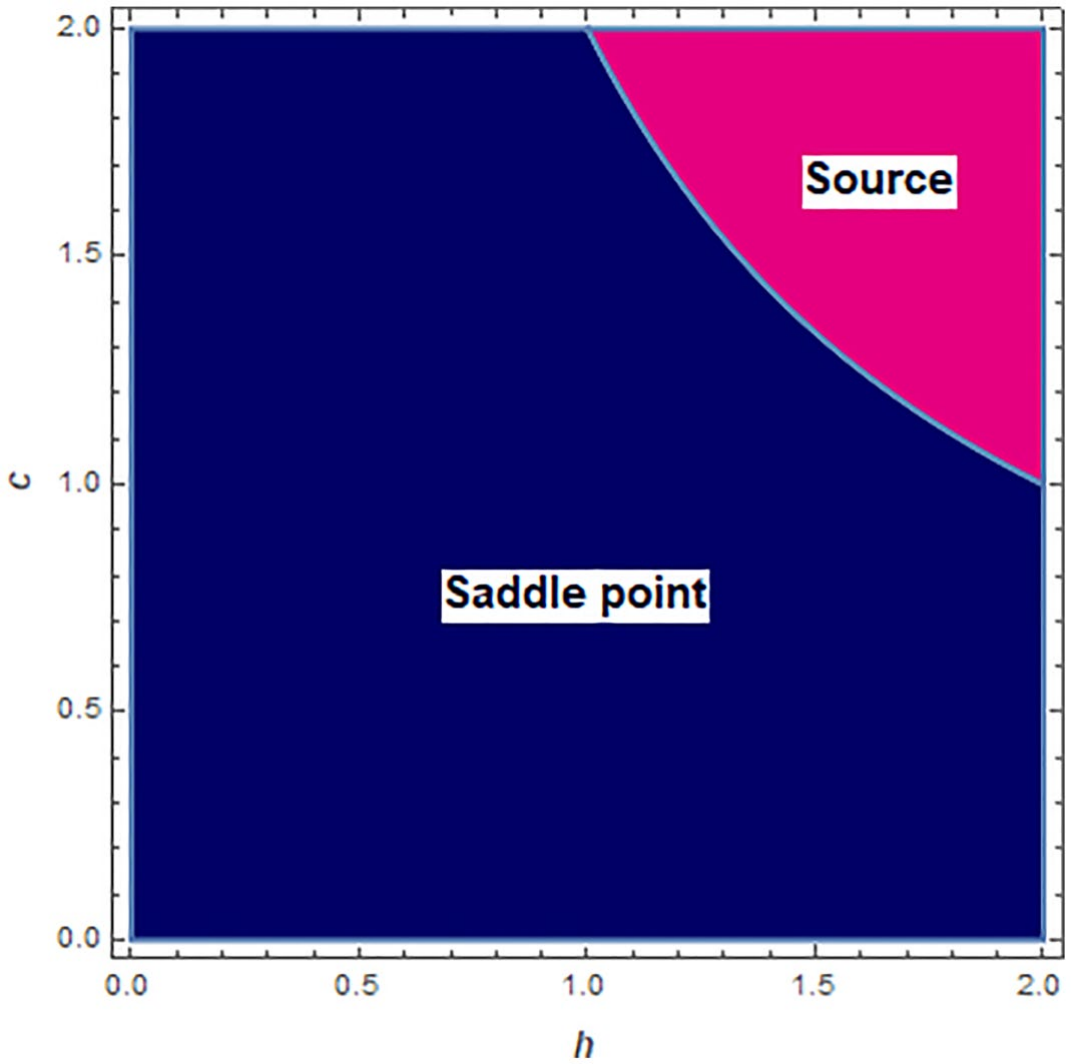
Topological classification of boundary fixed point $p_{0}(0,0)$ at $h\in \left[ 0,2\right]$ and $c\in \left[ 0,2\right]$.

From Theorem 1, when $h=\frac{2}{c}$, we observe that one of the eigenvalues around the fixed point $p_{0}(0,0)$ is $-1$. So, a flip bifurcation may happen when the parameter converts in the small neighborhood of $h=\frac{2}{c}$. For $h\in \left[ 0,2\right]$ and $c\in \left[ 0,2\right]$ topological classification of boundary fixed point $p_{0}(0,0)$ is depicted in Fig. 1. $\square$

### Theorem 2

When b > 2c, then the following statements hold true:

(i) If one set of the following conditions are true, then $p_{1}(\frac{ce}{\sqrt{(b-c)ce}},\frac{ae}{\sqrt{(b-c)ce}})$ is locally asymptotically stable (sink):
(ii) $$(b-2c)^{2}a-8bc(b-c)\ge 0$$ and $$0<h<\frac{(b-2c)a-\sqrt{ a((b-2c)^{2}a-8bc(b-c))}}{2ac(b-c)}$$.
(iii) $$(b-2c)^{2}a-8bc(b-c)<0$$ and $$0<h<\frac{b-2c}{2c(b-c)}$$.
(iv) If one set of the following conditions are true, then $p_{1}(\frac{ce}{\sqrt{ (b-c)ce}},\frac{ae}{\sqrt{(b-c)ce}})$ is unstable (source):
(v) $$(b-2c)^{2}a-8bc(b-c)\ge 0$$ and $$h>\frac{(b-2c)a+\sqrt{ a((b-2c)^{2}a-8bc(b-c))}}{2ac(b-c)}$$.
(vi) $$(b-2c)^{2}a-8bc(b-c)<0$$ and $$h>\frac{b-2c}{2c(b-c)}$$.
(vii) If one set of the following conditions are true, then $p_{1}(\frac{ce}{\sqrt{ (b-c)ce}},\frac{ae}{\sqrt{(b-c)ce}})$ is unstable (non-hyperbolic):
(viii) $(b-2c)^{2}a-8bc(b-c)\ge 0$ and $h=\frac{(b-2c)a\pm \sqrt{ a((b-2c)^{2}a-8bc(b-c))}}{2ac(b-c)}$, and $h\ne \frac{2b}{a\left( b-2c\right) }$, $\frac{4b}{a\left( b-2c\right) }$
(ix) $$(b-2c)^{2}a-8bc(b-c)<0$$ and $$h=\frac{b-2c}{2c(b-c)}$$.
(x) the fixed point $p_{1}(\frac{ce}{\sqrt{ (b-c)ce}},\frac{ae}{\sqrt{(b-c)ce}})$ is unstable (saddle point) if
(xi) $$(b-2c)^{2}a-8bc(b-c)>0$$,
(xii) $$\frac{(b-2c)a+\sqrt{a((b-2c)^{2}a-8bc(b-c))}}{2ac(b-c)}<h<\frac{(b-2c)a-\sqrt{ a((b-2c)^{2}a-8bc(b-c))}}{2ac(b-c)}$$.

### Proof

The Jacobian matrix at $p_{1}(\frac{ce}{\sqrt{(b-c)ce}},\frac{ae}{\sqrt{ (b-c)ce}})$ which has the form

$$\begin{aligned} J(x^{*},y^{*})=\left[ \begin{array}{cc} j_{11} &{} j_{12} \\ &{} \\ j_{21} &{} j_{22} \end{array} \right] , \end{aligned}$$

where

$$\begin{aligned} j_{11}=1-\dfrac{ah(b-2c)}{b},\text { }j_{12}=-hc,\text { }j_{21}=\dfrac{ 2ah(b-c)}{b}\text { and }j_{22}=1. \end{aligned}$$

Let

$$\begin{aligned} \mathcal {F}(R)=R^{2}-TR+D, \end{aligned}$$

where

$$\begin{aligned} \begin{array}{cc} T=j_{11}+j_{22}\,\text { and }\,D=-j_{12}j_{21}+j_{11}j_{22}, \end{array} \end{aligned}$$

then, we get

8 $$\begin{aligned} \mathcal {F}(-1)=4+2ah(hc-1)+\frac{2ah(2c-hc^{2})}{b}. \end{aligned}$$

From Lemma 1, we say that the fixed point is locally asymptotically stable If and only if $D<1$ and $\mathcal {F}(-1)>0$ and the fixed point is non hyperbolic if and only if $T\ne 0,2$ and $\mathcal {F}(-1)=0$. The results are therefore obtained by calculating (8).This completes our proof.

From Theorem 2, it is clear that one of the eigenvalues related to the unique positive equilibrium point is $p_{1}(\frac{ce}{\sqrt{(b-c)ce}},\frac{ ae}{\sqrt{(b-c)ce}})$ is $-1$ and the other is neither 1 nor $-1$ if (iii-1) in Theorem 2 holds. When (iii-2) in Theorem 2 is true, the eigenvalues related to $p_{1}(\frac{ ce}{\sqrt{(b-c)ce}},\frac{ae}{\sqrt{(b-c)ce}})$ are two conjugate complex numbers with the same modulus.

Let

$$\begin{aligned} F_{1P_{1}}=\left\{ \begin{array}{c} (a,b,c,e,h):h=h_{1}=\frac{(b-2c)a-\sqrt{a((b-2c)^{2}a-8bc(b-c))}}{2ac(b-c)}, b>2c \ and \ a(b-2c)>8bc \end{array} \right\} , \end{aligned}$$

and

$$\begin{aligned} F_{2P_{1}}=\left\{ \begin{array}{c} (a,b,c,e,h):h=h_{1}^{^{\prime }}=\frac{(b-2c)a+\sqrt{a((b-2c)^{2}a-8bc(b-c))} }{2ac(b-c)}, b>2c \ and \ a(b-2c)>8bc \end{array} \right\} . \end{aligned}$$

Then the unique positive equilibrium point $p_{1}(\frac{ce}{\sqrt{(b-c)ce}}, \frac{ae}{\sqrt{(b-c)ce}})$ may undergo the Flip bifurcation (period-doubling bifurcation) when the parameters vary in a small neighborhood of $F_{1P_{1}}$ or $F_{2P_{1}}$. Let

$$\begin{aligned} H_{P_{1}}=\left\{ \begin{array}{c} (a,b,c,e,h):h=h_{2}=\frac{b-2c}{2c(b-c)}\ and \ b>2c \end{array} \right\} . \end{aligned}$$

Then the unique positive equilibrium point $p_{1}(\frac{ce}{\sqrt{(b-c)ce}}, \frac{ae}{\sqrt{(b-c)ce}})$ may undergo the Neimark-Sacker bifurcation (hopf bifurcation) when the parameters vary in a small neighborhood of $H_{P_{1}}$.

## Bifurcations analysis

This section deals with the positive fixed point $p_{1}(\frac{ce}{\sqrt{ (b-c)ce}},\frac{ae}{\sqrt{(b-c)ce}})$ where, using the center manifold theorem and bifurcation theory, we define the integral step size *h* as a bifurcation parameter to investigate the flip bifurcation and Neimark-Sacker bifurcation of $p_{1}(\frac{ce}{\sqrt{(b-c)ce}},\frac{ae}{\sqrt{(b-c)ce}} )$.

### Flip bifurcation

Here we investigate the flip bifurcation of the discrete-time model (3) with respect to the unique positive fixed point $p_{1}(\frac{ce}{\sqrt{(b-c)ce}}, \frac{ae}{\sqrt{(b-c)ce}})$, when the parameters vary in a small neighborhood of $F_{1P_{1}}$ (similar arguments can be applied to the case of $F_{2P_{1}}$).

Taking the parameters (*a*, *b*, *c*, *e*, *h*) arbitrarily from $F_{1P_{1}}$, we consider System (3) with $(a,b,c,e,h)\in F_{1P_{1}}$ described by

9 $$\begin{aligned} \left\{ \begin{array}{c} x_{n+1}=x_{n}+h_{1}\left( ax_{n}-\frac{bx_{n}^{2}y_{n}}{e+x_{n}^{2}}\right) , \\ y_{n+1}=y_{n}-h_{1}\left( cy_{n}+\frac{bx_{n}^{2}y_{n}}{e+x_{n}^{2}}\right) . \end{array} \right. \end{aligned}$$

From Eq. (6), it is easy to obtain that the eigenvalues related to $p_{1}(\frac{ce}{\sqrt{(b-c)ce}},\frac{ae}{\sqrt{(b-c)ce}})$ are $R_{1}=-1$ and $R_{2}=3-\frac{h_{1}(b-2c)a}{b}$ with $\left| R_{2}\right| \ne 1$ by Theorem 2.

Choosing $h_{*}$ as a bifurcation parameter, we consider a perturbation of (9) as follows:

10 $$\begin{aligned} \left\{ \begin{array}{c} x_{n+1}=x_{n}+(h_{1}+h_{*})\left( ax_{n}-\frac{bx_{n}^{2}y_{n}}{e+x_{n}^{2}} \right) , \\ y_{n+1}=y_{n}-(h_{1}+h_{*})\left( cy_{n}+\frac{bx_{n}^{2}y_{n}}{e+x_{n}^{2}}\right) , \end{array} \right. \end{aligned}$$

where $\left| h_{*}\right|<<1$ is a small disturbance parameter.

Assume that $u=x-x^{*}$, $u=y-y^{*}$. Then we transform the fixed point $p_{1}(\frac{ce}{\sqrt{(b-c)ce}},\frac{ae}{\sqrt{(b-c)ce}})$ or $p_{1}(x^{*},y^{*})$ of System (10) into the origin. Then we have

11 $$\left( {\begin{array}{*{20}c} u \\ {} \\ v \\ \end{array} } \right) \to \left( {\begin{array}{*{20}c} \begin{gathered} \hat{E}_{{11}} u + \hat{E}_{{12}} v + \hat{E}_{{13}} uv + \hat{E}_{{14}} u^{2} \hfill \\ + \hat{P}_{1} uh_{*} + \hat{P}_{2} vh_{*} + \hat{P}_{3} uvh_{*} + \hat{P}_{4} u^{2} h_{*} \hfill \\ \end{gathered} \\ {} \\ \begin{gathered} \hat{E}_{{21}} u + \hat{E}_{{22}} v + \hat{E}_{{23}} uv + \hat{E}_{{24}} u^{2} \hfill \\ + \hat{P}_{5} uh_{*} + \hat{P}_{6} vh_{*} + \hat{P}_{7} uvh_{*} + \hat{P}_{8} u^{2} h_{*} \hfill \\ \end{gathered} \\ \end{array} } \right),$$

where

12 $$\begin{aligned} \begin{array}{lll} \hat{E}_{11}=ah+1-\frac{2hbex^{*}y^{*}}{\left( x^{*2}+e\right) ^{2}}, &{} \hat{E}_{12}=-\frac{bhx^{*2}}{x^{*2}+e}, &{} \hat{E}_{13}=- \frac{2hbex^{*}}{\left( x^{*2}+e\right) ^{2}}, \\ &{} &{} \\ \hat{E}_{14}=-\frac{hbey^{*}\left( -3x^{*2}+e\right) }{\left( x^{*2}+e\right) ^{3}}, &{} \hat{P}_{1}=a-\frac{2bex^{*}y^{*}}{ \left( x^{*2}+e\right) ^{2}}, &{} \hat{P}_{2}=-\frac{bx^{*2}}{x^{*2}+e}, \\ &{} &{} \\ \hat{P}_{3}=-\frac{2bex^{*}}{\left( x^{*2}+e\right) ^{2}}, &{} \hat{P} _{4}=-\frac{bey^{*}\left( -3x^{*2}+e\right) }{\left( x^{*2}+e\right) ^{3}}, &{} \\ &{} &{} \\ \hat{E}_{21}=\frac{2hbex^{*}y^{*}}{\left( x^{*2}+e\right) ^{2}}, &{} \hat{E}_{22}=1-ch+\frac{bhx^{*2}}{x^{*2}+e}, &{} \hat{E}_{23}=\frac{ 2hbex^{*}}{\left( x^{*2}+e\right) ^{2}}, \\ &{} &{} \\ \hat{E}_{24}=\frac{hbey^{*}\left( -3x^{*2}+e\right) }{\left( x^{*2}+e\right) ^{3}}, &{} \hat{P}_{5}=\frac{2bex^{*}y^{*}}{\left( x^{*2}+e\right) ^{2}}, &{} \hat{P}_{6}=\frac{bx^{*2}}{x^{*2}+e}-c, \\ &{} &{} \\ \hat{P}_{7}=\frac{2bex^{*}}{\left( x^{*2}+e\right) ^{2}} \ and \ \hat{P} _{8}=\frac{bey^{*}\left( -3x^{*2}+e\right) }{\left( x^{*2}+e\right) ^{3}}, &{} \end{array} \end{aligned}$$

and $$h=h_{1}.$$

The invertible matrix $\mathcal {M}$ defined by

$$\begin{aligned} \mathcal {M}=\left( \begin{array}{cc} \hat{E}_{12} &{} \hat{E}_{12} \\ -1-\hat{E}_{11} &{} k_{2}-\hat{E}_{11} \end{array} \right) , \end{aligned}$$

and apply the translation $(x,y)^{T}=\mathcal {M}(\bar{x},\bar{y})^{T}$. Then Map (11) may be changed into

13 $$\begin{aligned} \left( \begin{array}{c} x \\ \\ y \end{array} \right) \rightarrow \left( \begin{array}{cc} -1 &{} 0 \\ &{} \\ 0 &{} k_{2} \end{array} \right) \left( \begin{array}{c} \bar{x} \\ \\ \bar{y} \end{array} \right) +\left( \begin{array}{c} f(u,v,h_{*}) \\ \\ g(u,v,h_{*}) \end{array} \right) , \end{aligned}$$

where

14 $$\begin{aligned} f(u,v,h_{*})= & {} \dfrac{\left( \hat{E}_{14}\left( k_{2}-\hat{E}_{11}\right) -\hat{ E}_{12}\hat{E}_{24}\right) }{\hat{E}_{12}(1+k_{2})}u^{2}+\dfrac{\left( \hat{E}_{13}\left( k_{2}-\hat{E}_{11}\right) -\hat{E}_{23}\hat{E} _{12}\right) }{\hat{E}_{12}(1+k_{2})}uv\nonumber \\&+\dfrac{\left( \hat{P}_{4}\left( k_{2}-\hat{E} _{11}\right) -\hat{P}_{8}\hat{E}_{12}\right) }{\hat{E}_{12}(1+k_{2})}h_{*}u^{2} +\dfrac{\left( \hat{P}_{3}\left( k_{2}-\hat{E}_{11}\right) -\hat{P}_{7} \hat{E}_{12}\right) }{\hat{E}_{12}(1+k_{2})}h_{*}uv\nonumber \\&+\dfrac{\left( \hat{P}_{1}\left( k_{2}-\hat{E} _{11}\right) -\hat{P}_{5}\hat{E}_{12}\right) }{\hat{E}_{12}(1+k_{2})}h_{*}u+ \dfrac{\left( \hat{P}_{2}\left( k_{2}-\hat{E}_{11}\right) -\hat{P}_{6}\hat{E}_{12}\right) }{\hat{E}_{12}(1+k_{2})}h_{*}v\nonumber \\&+o((\left| u\right| +\left| v\right| +\left| h_{*}\right| )^{4}),\nonumber \\ g(u,v,h_{*})= & {} \dfrac{\left( \hat{E}_{14}\left( 1+\hat{E}_{11}\right) + \hat{E}_{12}\hat{E}_{24}\right) }{\hat{E}_{12}(1+k_{2})}u^{2}+\dfrac{(\hat{E} _{13}\left( 1+\hat{E}_{11}\right) +\hat{E}_{12}\hat{E}_{23})}{\hat{E} _{12}(1+k_{2})}uv\nonumber \\&+\dfrac{\left( \hat{P}_{4}\left( 1+\hat{E} _{11}\right) +\hat{E}_{12}\hat{P}_{8}\right) }{\hat{E}_{12}(1+k_{2})}h_{*}u^{2}+ \dfrac{\left( \hat{P}_{3}\left( 1+\hat{E}_{11}\right) +\hat{E}_{12}\hat{P}_{7}\right) }{ \hat{E}_{12}(1+k_{2})}h_{*}uv\nonumber \\&+\dfrac{\left( \hat{P}_{1}\left( 1+\hat{E} _{11}\right) +\hat{E}_{12}\hat{P}_{5}\right) }{\hat{E}_{12}(1+k_{2})}h_{*}u+ \dfrac{(\hat{P}_{2}\left( 1+\hat{E}_{11}\right) +\hat{E}_{12}\hat{P}_{6})}{ \hat{E}_{12}(1+k_{2})}h_{*}v\nonumber \\&+o((\left| u\right| +\left| v\right| +\left| h_{*}\right| )^{4}), \end{aligned}$$

with $$u=$$ $$\hat{E}_{12}\bar{x}+\hat{E}_{12}\bar{y}$$ and $$v=-(1+\hat{E}_{11})\bar{ x}+(k_{2}-\hat{E}_{11})\bar{y}$$.

Next, the center manifold theorem is then applied see^21^ to determine the dynamics of the fixed point $(\bar{x},\bar{y})=(0,0)$ at $h_{*}=0$. Then there exists a center manifold $W^{c}(0,0)$ of Map (13). It may be expressed as follows:

$$\begin{aligned} W^{c}(0,0)=\{(\bar{x},\bar{y}):\bar{y}=c_{1}\bar{x}^{2}+c_{2}\bar{x}h_{*}+c_{3}h_{*}^{2}+o((\left| \bar{x}\right| +\left| h_{*}\right| )^{3}\}, \end{aligned}$$

where $o((\left| \bar{x}\right| +\left| h_{*}\right| )^{3})$ is a function with at least three orders in its variables $(\bar{x},h_{*})$

and

$$\begin{aligned} c_{1}= & {} \frac{\hat{E}_{12}^{2}\hat{E}_{24}+(1+\hat{E}_{11})(\hat{E}_{14}-\hat{E }_{23})\hat{E}_{12}-\hat{E}_{13}(1+\hat{E}_{11})^{2}}{\left( 1-k_{2}^{2}\right) },\\ c_{2}= & {} \frac{\hat{P}_{2}(1+\hat{E}_{11})^{2}-\hat{E}_{12}^{2}\hat{P} _{5}-(1+\hat{E}_{11})(\hat{P}_{1}-\hat{P}_{6})\hat{E}_{12}}{\hat{E} _{12}\left( 1+k_{2}\right) ^{2}}, \\ c_{3}= & {} 0. \end{aligned}$$

Therefore, Map (14) restricted to $W^{c}(0,0)$ is given by

$$\begin{aligned} F:\bar{x}\rightarrow -\bar{x}+s_{1}\bar{x}^{2}+s_{2}\bar{x}h_{*}+s_{3} \bar{x}^{2}h_{*}+s_{4}\bar{x}h_{*}^{2}+s_{5}\bar{x}^{3}+O((|\bar{x} |+|h_{*}|^{4}), \end{aligned}$$

where

$$\begin{aligned} s_{1}= & {} \frac{1}{k_{2}+1}(\hat{E}_{11}^{2}\hat{E}_{13}+\left( \left( -\hat{E} _{14}+\hat{E}_{23}\right) \hat{E}_{12}-\hat{E}_{13}(-1+k_{2})\right) \hat{E} _{11}\\&-\hat{E}_{12}^{2}\hat{E}_{24}+(\hat{E}_{14}k_{2}+\hat{E} _{23})\hat{E}_{12}-k_{2}\hat{E}_{13}), \\ s_{2}= & {} \frac{1}{\hat{E}_{12}\left( k_{2}+1\right) }((\hat{E}_{11}(\hat{P}_{6}- \hat{P}_{1})+k_{2}\hat{P}_{1}+\hat{P}_{6})\hat{E}_{12}-\hat{P}_{2}(1+\hat{E} _{11})\left( k_{2}-\hat{E}_{11}\right) \\&-\hat{E}_{12}^{2}\hat{P}_{5}), \\ s_{3}= & {} \frac{1}{\hat{E}_{12}\left( k_{2}+1\right) }(-\hat{E}_{12}^{3}\left( 2 \hat{E}_{24}c_{2}+\hat{P}_{8}\right) +(\left( \left( 2\hat{E}_{23}-2\hat{E} _{14}\right) c_{2}-\hat{P}_{4}+\hat{P}_{7}\right) \hat{E}_{11} \\&+\left( \left( 2\hat{E}_{14}-\hat{E}_{23}\right) c_{2}+ \hat{P}_{4}\right) k_{2}-c_{1}\hat{P}_{5}+c_{2}\hat{E}_{23}+\hat{P}_{7})\hat{ E}_{12}^{2} \\&+\left( k_{2}-\hat{E}_{11}\right) ((-2\hat{E}_{13}c_{2}- \hat{P}_{3})\hat{E}_{11}+\hat{E}_{13}c_{2}k_{2}-\hat{E}_{13}c_{2} \\&+(\hat{P}_{1}-\hat{P}_{6})c_{1}-\hat{P}_{3})\hat{E} _{12}+c_{1}\hat{P}_{2}(k_{2}-\hat{E}_{11})^{2}), \\ s_{4}= & {} \frac{1}{\hat{E}_{12}\left( k_{2}+1\right) }((-\hat{E}_{12}^{2}\hat{P} _{5}+(\hat{P}_{1}-\hat{P}_{6})\left( k_{2}-\hat{E}_{11}\right) \hat{E}_{12}+ \hat{P}_{2}(k_{2}-\hat{E}_{11})^{2})c_{2}), \end{aligned}$$

and

$$\begin{aligned} s_{5}= & {} \frac{1}{\left( k_{2}+1\right) }((-2\hat{E}_{12}^{2}\hat{E} _{24}+(\left( 2\hat{E}_{14}-\hat{E}_{23}\right) k_{2}+\left( 2\hat{E}_{23}-2 \hat{E}_{14}\right) \hat{E}_{11} \\&+\hat{E}_{23})\hat{E}_{12}+\hat{E}_{13}(k_{2}-\hat{E} _{11})(k_{2}-2\hat{E}_{11}-1))c_{1}). \end{aligned}$$

Let

$$\begin{aligned} \Gamma _{1}=\left( \frac{\partial ^{2}F}{\partial \bar{x}\partial h_{*}}+ \frac{1}{2}\frac{\partial F}{\partial h_{*}}\frac{\partial ^{2}F}{ \partial \bar{x}^{2}}\right) |_{\left( 0,0\right) }=s_{2,} \end{aligned}$$

and

$$\begin{aligned} \Gamma _{2}=\left( \frac{1}{6}\frac{\partial ^{3}F}{\partial \bar{x}^{3}}+( \frac{1}{2}\frac{\partial ^{2}F}{\partial \bar{x}^{2}})^{2}\right) |_{\left( 0,0\right) }=s_{1}^{2}+s_{5}. \end{aligned}$$

From the previous discussion, we get the following theorem:

#### Theorem 3

If $\Gamma _{1}\ne 0$ and $\Gamma _{2}\ne 0$, then System ( 3) undergoes a flip bifurcation at the unique positive fixed point $p_{1}(\frac{ce}{\sqrt{(b-c)ce}},\frac{ae}{\sqrt{(b-c)ce}})$ when the parameter *h* varies in a small neighborhood of $F_{P_{1}}$. Moreover, if $\Gamma _{2}>0$ (resp., $\Gamma _{2}<0$), then the period-2 orbits that bifurcate from $p_{1}(\frac{ce}{\sqrt{(b-c)ce}},\frac{ae}{\sqrt{(b-c)ce}})$ are stable (resp., unstable).

### Neimark-Sacker bifurcation

Next, we discuss the Neimark-Sacker bifurcation of $p_{1}(\frac{ce}{\sqrt{ (b-c)ce}},\frac{ae}{\sqrt{(b-c)ce}})$ when the parameters (*a*, *b*, *c*, *e*, *h*) vary in a small neighborhood of $H_{p_{1}}$. We consider System (3) with$(a,b,c,e,h)\in H_{p_{1}}$ represented by

15 $$\begin{aligned} \left\{ \begin{array}{c} x_{n+1}=x_{n}+h_{2}(ax_{n}-\frac{bx_{n}^{2}y_{n}}{e+x_{n}^{2}}), \\ y_{n+1}=y_{n}-h_{2}(cy_{n}+\frac{bx_{n}^{2}y_{n}}{e+x_{n}^{2}}). \end{array} \right. \end{aligned}$$

Then Map (15) has a unique positive fixed point $p_{1}(\frac{ce}{ \sqrt{(b-c)ce}},\frac{ae}{\sqrt{(b-c)ce}})$.

Then we choose $\bar{h}_{*}$ as a bifurcation parameter and consider a perturbation of (15) as follows:

16 $$\begin{aligned} \left\{ \begin{array}{l} x_{n+1}=x_{n}+(h_{2}+\bar{h}_{*})(ax_{n}-\frac{bx_{n}^{2}y_{n}}{ e+x_{n}^{2}}), \\ y_{n+1}=y_{n}+(h_{2}+\bar{h}_{*})\left( cy_{n}+\frac{bx_{n}^{2}y_{n}}{ e+x_{n}^{2}}\right) , \end{array} \right. \end{aligned}$$

where $\left| \bar{h}_{*}\right|<<1$ is a small perturbation parameter.

Assume that $u=x-x^{*}$, $u=y-y^{*}$. Then we transform the fixed point $p_{1}(\frac{ce}{\sqrt{(b-c)ce}},\frac{ae}{\sqrt{(b-c)ce}})$ of Map (10) into the origin. Then we have

17 $$\begin{aligned} \left( \begin{array}{c} u \\ \\ v \end{array} \right) \rightarrow \left( \begin{array}{c} \hat{E}_{11}u+\hat{E}_{12}v+\hat{E}_{13}uv+\hat{E}_{14}u^{2} \\ \\ \hat{E}_{21}u+\hat{E}_{22}v+\hat{E}_{23}uv+\hat{E}_{24}u^{2} \end{array} \right) , \end{aligned}$$

where $\hat{E}_{11},$ $\hat{E}_{12},$ $\hat{E}_{13},$ $\hat{E}_{14},$ $\hat{E }_{21},$ $\hat{E}_{22},$ $\hat{E}_{23},$ $\hat{E}_{24}$ are given in (14) by substituting *h* for $h_{2}+\bar{h}_{*}$.

Then Map (15) has a unique positive fixed point $p_{1}(x^{*},y^{*})$, where $x^{*}=\frac{ce}{\sqrt{(b-c)ce}}$, $y^{*}=\frac{ae}{\sqrt{(b-c)ce}}$.

Then the characteristic equation model (16) at $p_{1}(x^{*},y^{*})$ is given by

$$\begin{aligned} R^{2}-p(\bar{h}_{*})R+q(\bar{h}_{*})=0, \end{aligned}$$

where

$$\begin{aligned} p(\bar{h}_{*})=2+\frac{a\left( h_{2}+\bar{h}_{*}\right) (2c-b)}{b}, \end{aligned}$$

and

$$\begin{aligned} q(\bar{h}_{*})=1+\frac{2ac\left( h_{2}+\bar{h}_{*}\right) ^{2}(b-c)}{ b}+\frac{a\left( h_{2}+\bar{h}_{*}\right) (2c-b)}{b}. \end{aligned}$$

Since parameters $(a,b,c,e,h)\in H_{p_{1}}$, the eigenvalues of $p_{1}(x^{*},y^{*})$ are a pair of complex conjugate numbers *R*,  $\overline{R}$ with modulus 1 by Theorem 2, where

$$\begin{aligned} R,\overline{R}= & {} -\frac{p(\bar{h}_{*})}{2}\pm i\frac{\sqrt{4q(\bar{h} _{*})-p^{2}(\bar{h}_{*})}}{2}, \\= & {} \frac{2b-a(h_{2}+\bar{h}_{*})(b-2c)}{2b} \\&\frac{\pm i(h_{2}+\bar{h}_{*})\sqrt{a(8bc(b-c)-a(b-2c)^{2})}}{2b} \end{aligned}$$

Then we have

$$\begin{aligned} \left| R\right| =\sqrt{q(\bar{h}_{*})},\ell =\frac{d\left| R\right| }{d\bar{h}_{*}}|_{\bar{h}_{*}=0}=\frac{a(b-2c)}{2b}>0. \end{aligned}$$

In addition, we require that when $\bar{h}_{*}=0$, $R^{n}$, $\overline{R} ^{n}\ne 1$, $n=1,$ 2,  3,  4, which is equivalent to $p(0)\ne -2,$ 0,  1,  2. Note that $(a,b,c,e,h)\in H_{p_{1}}$, so $p(0)\ne -2,$ 2. Thus we only need to satisfy $p(0)\ne 0,$ 1, which leads to

18 $$\begin{aligned} a(b-2c)^{2}\ne 4bc(b-c),6bc(b-c). \end{aligned}$$

In the following, we investigate the normal form of Map (17) at $\bar{h}_{*}=0$.

Put

$$\begin{aligned} m=1-\frac{ah(b-2c)}{2b}, \end{aligned}$$

and

$$\begin{aligned} \omega =\frac{h\sqrt{a(8bc(b-c)-a(b-2c)^{2})}}{2b}. \end{aligned}$$

Using the translation

$$\begin{aligned} \left( \begin{array}{c} u \\ \\ v \end{array} \right) =\left( \begin{array}{cc} \hat{E}_{12} &{} 0 \\ &{} \\ m-\hat{E}_{11} &{} -\omega \end{array} \right) \left( \begin{array}{c} X \\ \\ Y \end{array} \right) , \end{aligned}$$

the model (17) becomes

19 $$\begin{aligned} \left( \begin{array}{c} X \\ \\ Y \end{array} \right) \rightarrow \left( \begin{array}{cc} m &{} -\omega \\ &{} \\ \omega &{} m \end{array} \right) \left( \begin{array}{c} X \\ \\ Y \end{array} \right) +\left( \begin{array}{c} f(X,Y,h_{*}) \\ \\ g(X,Y,h_{*}) \end{array} \right) , \end{aligned}$$

where

$$\begin{aligned} \check{f}(X,Y,h_{*})= & {} \dfrac{1}{\hat{E}_{12}}(\hat{E}_{13}uv+\hat{E} _{14}u^{2}), \\ \check{g}(X,Y,h_{*})= & {} \dfrac{((m-\hat{E}_{11})\hat{E}_{14}-\hat{E}_{12} \hat{E}_{24})u^{2}}{\hat{E}_{12}\omega }\\&+\dfrac{((m-\hat{E}_{11})\hat{E} _{13}-\hat{E}_{12}\hat{E}_{23})uv}{\hat{E}_{12}\omega }. \end{aligned}$$

In addition,

$$\begin{aligned}{}&\begin{array}{ccc} \check{f}_{XX}=2(m-\hat{E}_{11})\hat{E}_{13}+2\hat{E}_{12}\hat{E}_{14},&\check{f}_{XY}=-\omega \hat{E}_{13},&\check{f}_{YY}=0, \end{array} \\&\\&\begin{array}{cccc} \check{f}_{XXX}=0,&\check{f}_{XXY}=0,&\check{f}_{XYY}=0,&\check{f} _{YYY}=0, \end{array} \end{aligned}$$

and

$$\begin{aligned} \begin{array}{l} \check{g}_{XX}=\dfrac{2}{\omega }((\hat{E}_{14}-\hat{E}_{23})(m-\hat{E}_{11}) \hat{E}_{12}+(m-\hat{E}_{11})^{2}\hat{E}_{13}-\hat{E}_{12}^{2}\hat{E}_{24}),\\ \\ \check{g}_{XY}=-(m-\hat{E}_{11})\hat{E}_{13}+\hat{E}_{12}\hat{E}_{23}, \\ \\ \check{g}_{YY}=0, \begin{array}{cccc} \check{g}_{XXX}=0, &{} \check{g}_{XXY}=0, &{} \check{g}_{XYY}=0, &{} \check{g} _{YYY}=0. \end{array} \end{array} \end{aligned}$$

Then Map (19) can undergo the Neimark-Sacker bifurcation when the following discriminatory quantity is not zero:

$$\begin{aligned} \Theta =Re[\frac{(1-2R) \bar{R}^{2}}{1-R}\Phi _{11} \Phi _{20} ]+ \frac{1}{2}\left| \Phi _{11} \right| ^{2}+\left| \Phi _{02} \right| ^{2}-Re(R \Phi _{21}), \end{aligned}$$

where

$$\begin{aligned} \Phi _{20}= & {} \frac{1}{8}[\check{f}_{XX}-\check{f}_{YY}+2\check{g}_{XY}+i( \check{g}_{XX}-\check{g}_{YY}-2\check{f}_{XY})], \\ \Phi _{11}= & {} \frac{1}{4}[\check{f}_{XX}+\check{f}_{YY}+i(\check{g}_{XX}+ \check{g}_{YY})], \\ \Phi _{02}= & {} \frac{1}{8}[\check{f}_{XX}-\check{f}_{YY}-2\check{g}_{XY}+i( \check{g}_{XX}-\check{g}_{YY}+2\check{f}_{XY})], \\ \Phi _{21}= & {} \frac{1}{16}[\check{f}_{XXX}+\check{f}_{XYY}+\check{g} _{XXY}+\check{g}_{YYY}+i(\check{g}_{XXX}+\check{g}_{XYY}-\check{f}_{XXY}- \check{f}_{YYY})]. \end{aligned}$$

Based on this analysis and the Neimark-Sacker bifurcation theorem discussed in^22,23^, we arrive at the following theorem.

#### Theorem 4

If condition (18) holds and $\Theta \ne 0$, then System (3) undergoes a Neimark-Sacker bifurcation at the unique positive fixed point $p_{1}(\frac{ce}{\sqrt{(b-c)ce}},\frac{ae}{\sqrt{(b-c)ce}})$ when the parameter *h* varies in a small neighborhood of $H_{p_{1}}$. Furthermore, if $\Theta <0$ (resp., $\Theta >0$), then an attracting (resp., repelling) invariant closed curve bifurcates from the fixed point for $h>h_{2}$ (resp., $h<h_{2}$).

#### Remark 1

According to bifurcation theory discussed in^24^, the bifurcation is called a supercritical Neimark-Sacker bifurcation if the discriminatory quantity $\Theta <0$. In the following section, numerical simulations guarantee that a supercritical Neimark-Sacker bifurcation occurs for the discrete-time model (4).

## Chaos control

In this section, our goal is to apply a feedback control method known as Ott-Grebogi-Yorke (OGY)^25–27^, to System (3). For controlling chaos under the effect of Neimark-Sacker and Period-doubling bifurcation at positive fixed point of System (3). To apply the OGY method, we write System (3) as follows:

20 $$\begin{aligned} x_{n+1}= & {} (1+ah)x_{n}-\frac{hbx_{n}^{2}y_{n}}{e+x_{n}^{2}}=f(x_{n},y_{n},c), \nonumber \\ y_{n+1}= & {} (1-ch)y_{n}+\frac{hbx_{n}^{2}y_{n}}{e+x_{n}^{2}}=g(x_{n},y_{n},c), \end{aligned}$$

where *c* is taken for chaos control parameter. Furthermore, *c* it is assumed that $c\in \left( c_{0}-\delta ,c_{0}+\delta \right)$ with $\delta >0$ and $c_{0}$ denotes the nominal value of *c*. Moreover, we consider $p_{1}(x^{*},y^{*})=p_{1}(\frac{ce}{\sqrt{(b-c)ce}},\frac{ae}{\sqrt{ (b-c)ce}})$ as positive fixed point of System (3). Then, one can approximate System (20) in the neighborhood of the fixed point $p_{1}(x^{*},y^{*})=p_{1}(\frac{ce}{\sqrt{(b-c)ce}},\frac{ae}{\sqrt{ (b-c)ce}})$ as follows:

21 $$\begin{aligned} \left[ \begin{array}{c} x_{n+1}-x^{*} \\ y_{n+1}-y^{*} \end{array} \right] \approx J(x^{*},y^{*},c_{0})\left[ \begin{array}{c} x_{n}-x^{*} \\ y_{n}-y^{*} \end{array} \right] +C[c-c_{0}] , \end{aligned}$$

where

$$\begin{aligned} J(x^{*},y^{*},c_{0})=\left[ \begin{array}{cc} \frac{\partial f(x^{*},y^{*},c_{0})}{\partial x} &{} \frac{\partial f(x^{*},y^{*},c_{0})}{\partial y} \\ &{} \\ \frac{\partial g(x^{*},y^{*},c_{0})}{\partial x} &{} \frac{\partial g(x^{*},y^{*},c_{0})}{\partial y} \end{array} \right] , \end{aligned}$$

and

$$C = \left[ {\begin{array}{*{20}c} {\frac{{\partial f(x^{*} ,y^{*} ,c_{0} )}}{{\partial c}}} \\ {\frac{{\partial g(x^{*} ,y^{*} ,c_{0} )}}{{\partial c}}} \\ \end{array} } \right] = \left[ {\begin{array}{*{20}c} 0 \\ { - \frac{{ah\sqrt {(b - c_{0} )c_{0} e} }}{{(b - c_{0} )c_{0} }}} \\ \end{array} } \right],$$

Moreover, System (20) is controlled by the following matrix:

$$\begin{aligned} \check{T}=\left[ C:JC\right] =\left[ \begin{array}{ccc} \frac{\partial f(x^{*},y^{*},c_{0})}{\partial c} &{} &{} \frac{\partial f(x^{*},y^{*},c_{0})}{\partial x}\cdot \frac{\partial f(x^{*},y^{*},c_{0})}{\partial c} \\ &{} &{} \\ \frac{\partial g(x^{*},y^{*},c_{0})}{\partial c} &{} &{} \frac{\partial g(x^{*},y^{*},c_{0})}{\partial x}\cdot \frac{\partial g(x^{*},y^{*},c_{0})}{\partial c} \end{array} \right] , \end{aligned}$$

has rank 2. Since $\sqrt{(b-c_{0})c_{0}e}>0$, therefore rank of $\check{T}$ is 2. Next, we assume that $$[c-c_{0}]=-K\left[ \begin{array}{c} x_{n}-x^{*} \\ y_{n}-y^{*} \end{array} \right]$$, where $$K=\left[ \begin{array}{cc} \rho _{1}&\rho _{2} \end{array} \right]$$, then System (21) can be written as

$$\begin{aligned} \left[ \begin{array}{c} x_{n+1}-x^{*} \\ y_{n+1}-y^{*} \end{array} \right] \approx \left[ J-CK\right] \left[ \begin{array}{c} x_{n}-x^{*} \\ y_{n}-y^{*} \end{array}\right] . \end{aligned}$$

Furthermore, the positive fixed point $p_{1}(x^{*},y^{*})$ is locally asymptotically stable if and only if both eigenvalues of the matrix $J-CK$ lie in an open unit disk. Now the matrix $J-CK$ can be written as follows:

$$\begin{aligned} J-CK=\left[ \begin{array}{cc} j_{11} &{} j_{12} \\ &{} \\ -\Phi \rho _{1}+j_{12} &{} -\Phi \rho _{2}+j_{22} \end{array} \right] , \end{aligned}$$

where

$$\begin{aligned} j_{11}= & {} \dfrac{ah(2c-b)+b}{b}, \quad j_{12}=-hc, \\ j_{21}= & {} \dfrac{2ah(b-c)}{b}, \quad j_{22}=1,\\ \Phi= & {} -\dfrac{ah\sqrt{(b-c)ce}}{(b-c)c}. \end{aligned}$$

The characteristic equation of the Jacobian matrix $J-CK$ is given by

22 $$\begin{aligned} \varvec{\rho }(R)=R^{2}-(j_{11}+j_{22}-\Phi \rho _{1})R+j_{11}(j_{22}-\Phi \rho _{2})-j_{12}(j_{12}-\Phi \rho _{1}). \end{aligned}$$

Let $R_{1}$ and $R_{2}$ are the eigenvalues of characteristic Eq. (22), then we have

23 $$\begin{aligned} R_{1}+R_{2}=j_{11}+j_{22}-\Phi \rho _{1}, \end{aligned}$$

and

24 $$\begin{aligned} R_{1}R_{2}=j_{11}(j_{22}-\Phi \rho _{2})-j_{12}(j_{21}-\Phi \rho _{1}). \end{aligned}$$

Moreover, we take $R_{1}=\pm 1$ and $R_{1}R_{2}=1$. Thus, the lines of marginal stability for (23) and (24) are computed as follows:

25 $$\begin{aligned} H_{1}:j_{11}(j_{22}-\Phi \rho _{2})-j_{12}(j_{21}-\Phi \rho _{1})-1=0. \end{aligned}$$

Next, we suppose that $R_{1}=1$, then Eqs. (24) and (23) yield that:

26 $$\begin{aligned} H_{2}:j_{22}+j_{12}j_{21}+\Phi (j_{11}\rho _{2}-\rho _{1}(j_{12}+1))+j_{11}(1-j_{22})-1=0. \end{aligned}$$

Finally, if $R_{1}=-1$ and using equations (23) we get

27 $$\begin{aligned} H_{3}:j_{22}-j_{12}j_{21}+\Phi (\rho _{1}(j_{12}-1)-j_{11}\rho _{2})+j_{11}(1+j_{22})+1=0. \end{aligned}$$

Then, stability region for (20) is triangular region bounded by $H_{1},H_{2}$ and $H_{3}$ in $\rho _{1}\rho _{2}$-plane.

## Numerical simulations

In this section, we present bifurcation diagrams, phase images, and maximum Lyapunov (ML in short) exponents of System (3) in order to highlight our theoretical analysis and demonstrate complex dynamical behaviors using numerical simulation.

### Flip bifurcation

#### Example 1

**Figure 2:**
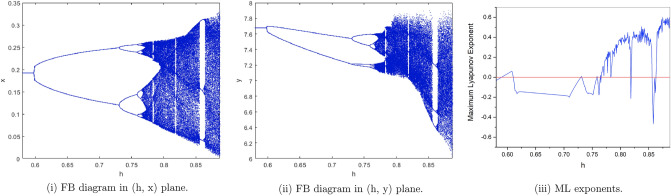
Bifurcation diagrams and ML exponents for the model (i) for values of a = 4, b = 2, c = 0.1, e = 0.7, h ϵ [0.58, 0.8875].

**Figure 3:**
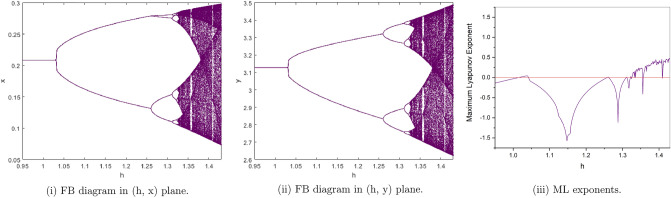
Bifurcation diagrams and ML exponents for the model (i) for values of a = 3, b = 2.5, c = 0.2, e = 0.5, h ϵ [0.95, 1.43].

*Case 1* We consider h as a parameter and consider the following subcases:

(*I*) $a=4, b=2, c=0.1, e=0.7$. We have only one positive fixed point. By calculation the flip bifurcation of model (3) shows from the fixed point $p_{1}(x^{*}, y^{*})=(0.191942974, 7.67771896)$ at $h=0.5926274349$ with $\Gamma _{1}=-3.374801576$, $\Gamma _{2}=0.2675441516$, and $(a,b,c,e,h)\in F_{P_{1}}$, which illustrates Theorem 3. From Fig. 2(i), (ii) we observe that the fixed point $p_{1}(x^{*}, y^{*})$ is stable for $0.58\le h<0.5926274349$ and loses its stability at the flip bifurcation parameter value $h=0.5926274349$. Also, there is a cascade of period -2, 4, 8, 16 orbits emerging. The maximum Lyapunov exponents corresponding to Fig. 2(i), (ii) are shown in Fig. 2(iii).

(*II*) $a=3, b=2.5, c=0.2, e=0.5$. By computation the flip bifurcation of model (3) Shows from the fixed point $p_{1}(x^{*}, y^{*})=(0.2085144141, 3.127716212)$ at $h=1.022801547$ with $\Gamma _{1}=-1.955413544$, $\Gamma _{2}=1.553116178$, and $(a,b,c,e,h)\in F_{P_{1}}$, which illustrates Theorem 3. From Fig. 3(i), (ii). we observe that the fixed point $p_{1}(x^{*},y^{*})$ is stable for $0.95\le h<1.022801547$ and loses its stability at the flip bifurcation parameter value $h=1.022801547$. Also, there is a cascade of period -2, 4, 8, 16 orbits emerging. The maximum Lyapunov exponents corresponding to Fig. 3(i ), (ii). are shown in Fig. 3(iii).

**Figure 4:**
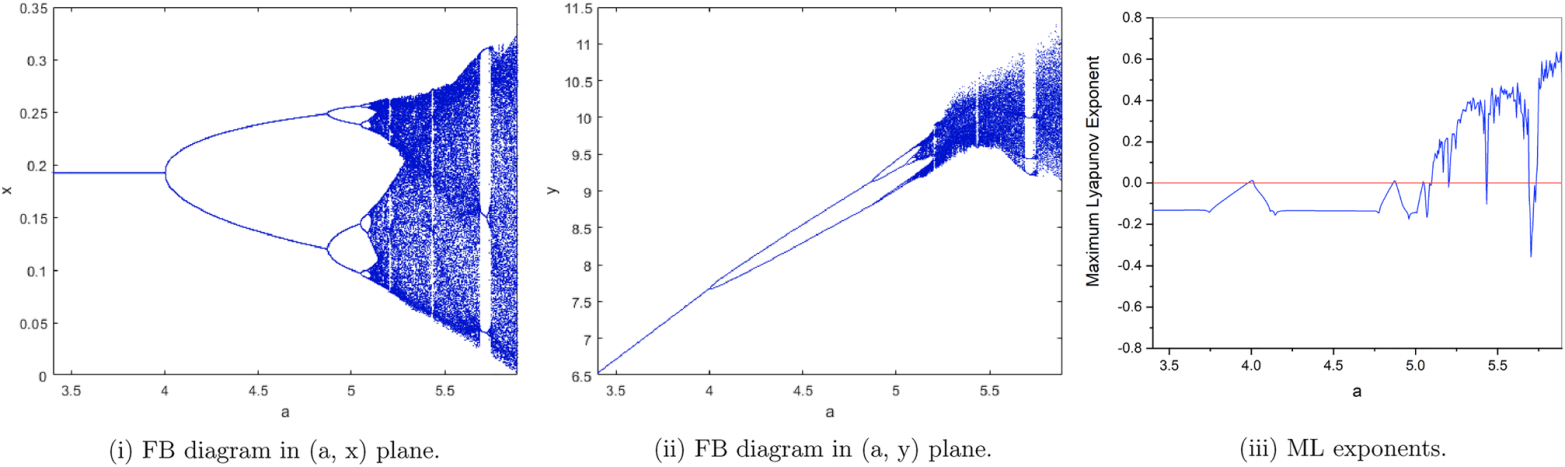
Bifurcation diagrams and ML exponents for the model (i) for values of b = 2, c = 0.1, e = 0.7, h = 0.5926274349, a ϵ [3.4, 5.89].

*Case 2* We consider a as a parameter and consider the following subcases:

$(I)^{'}$ $b=2, c=0.1, e=0.7, h=0.5926274349$. from Fig. 4(i), (ii) we observe that the fixed point $p_{1}(x^{*}, y^{*})$ is stable for $3.4\le a<4$ and loses its stability at the flip bifurcation parameter value $a=4$. The maximum Lyapunov exponents corresponding to Fig. 4(i), (ii) are shown in Fig. 4(iii).

**Figure 5:**
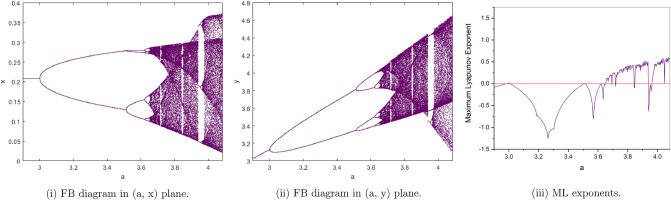
Bifurcation diagrams and ML exponents for the model (i) for values of b = 2.5, c = 0.2, e = 0.5, h = 1.022801547, a ϵ [2.9, 4.084].

$(II)^{'}$ $b=2.5,c=0.2,e=0.5,h=1.022801547$. from Fig. 5(i), (ii) we observe that the fixed point $p_{1}(x^{*}, y^{*})$ is stable for $2.9\le a<3$ and loses its stability at the flip bifurcation parameter value $a=3$. The maximum Lyapunov exponents corresponding to Fig. 5(i), (ii) are shown in Fig. 5(iii).

**Figure 6:**
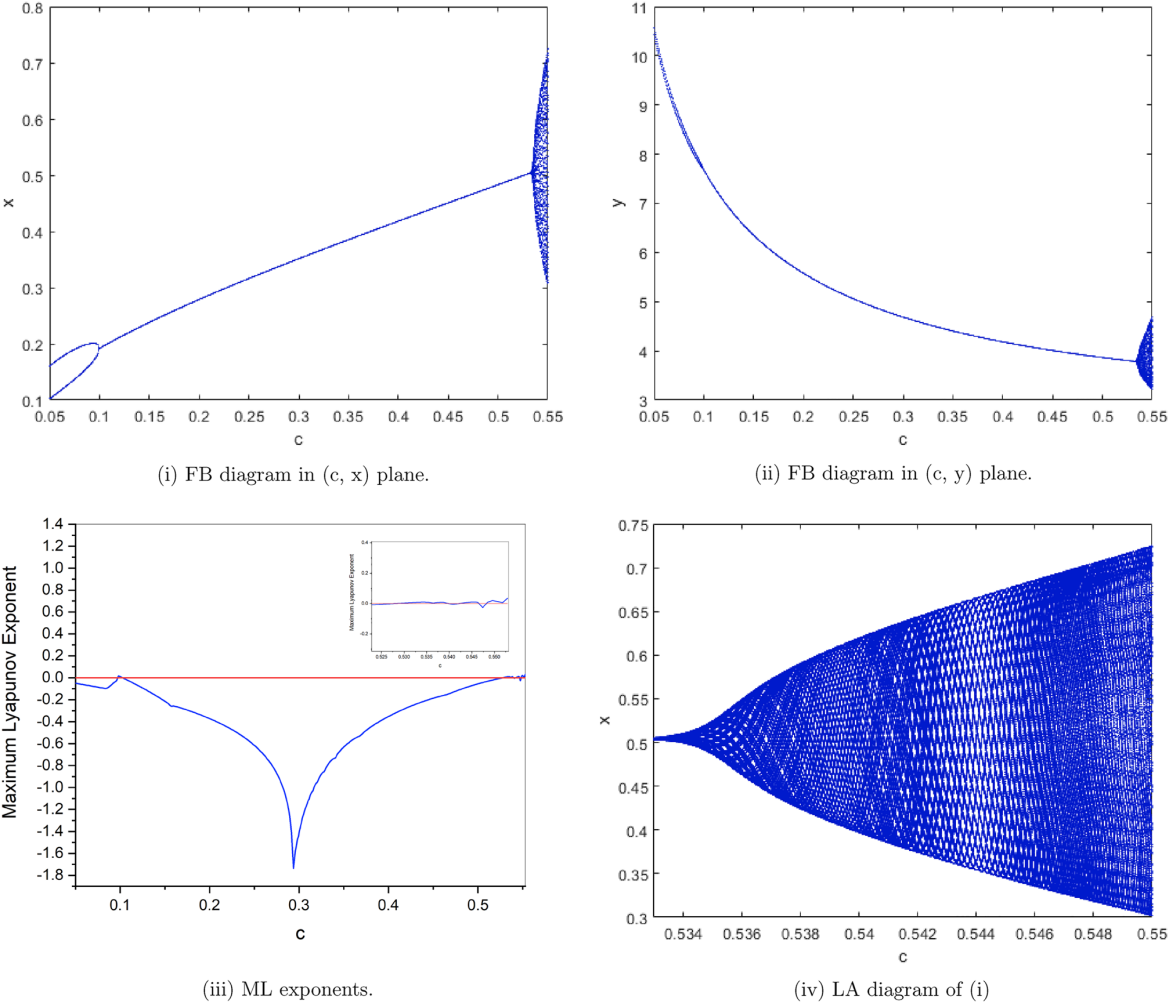
Bifurcation diagrams and ML exponents for the model (i) for values of a = 4, b = 2, e = 0.7, h = 0.5926274349, c ϵ [0.05, 0.5529] and LA corresponding to (i) for c ϵ [0.533, 0.55].

*Case 3* We consider c as a parameter and consider the following subcases:

$(I)^{''}$ $a=4, b=2, e=0.7, h=0.5926274349$. from Fig. 6(i), (ii) we observe that the fixed point $p_{1}(x^{*}, y^{*})$ is stable for $0.05\le c<0.1$ and loses its stability at the flip bifurcation parameter value $c=0.1$. The maximum Lyapunov exponents corresponding to Fig. 6(i), (ii) are shown in Fig. 6(iii). local amplification (LA in short) corresponding to Fig. 6(iv) for $0.533 \le c \le 0.55$ is shown in Fig. 6(i)

**Figure 7:**
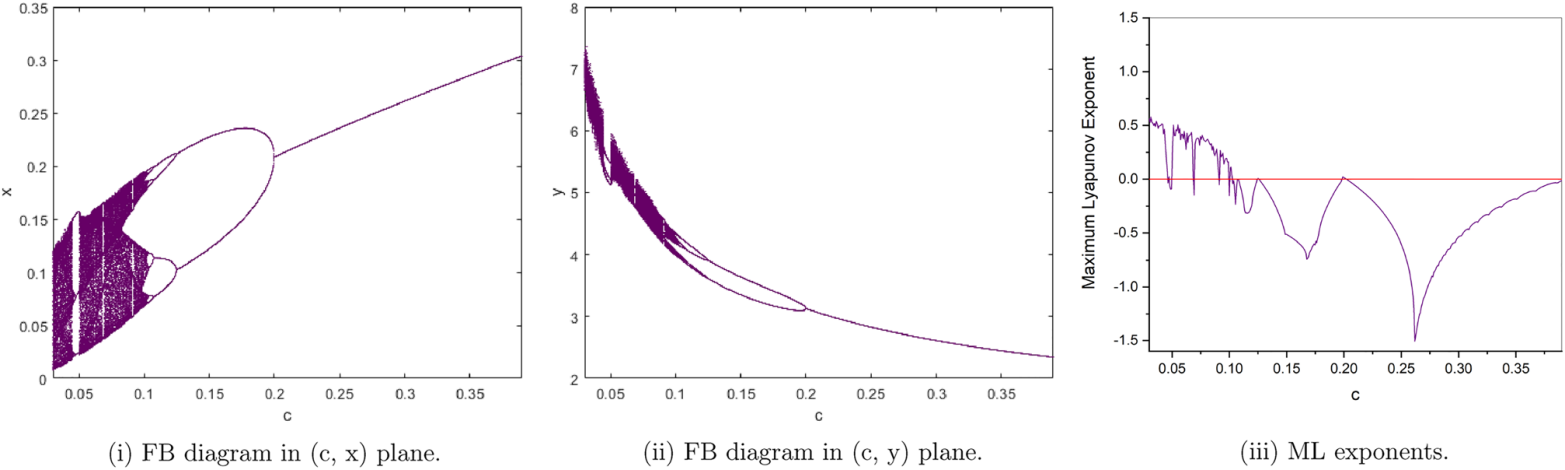
Bifurcation diagrams and ML exponents for the model (i) for values of a = 3, b = 2.5, e = 0.5, h = 1.022801547, c ϵ [0.03, 0.39].

$(II)^{''}$ $a=3, b=2.5, e=0.5,h=1.022801547$. from Fig. 7(i), (ii) we observe that the fixed point $p_{1}(x^{*}, y^{*})$ is stable for $0.2<c\le 0.39$ and loses its stability at the flip bifurcation parameter value $c=0.2$. The maximum Lyapunov exponents corresponding to Fig. 7(i), (ii) are shown in Fig. 7(iii).

### Neimark-Sacker bifurcation

#### Example 2

**Figure 8:**
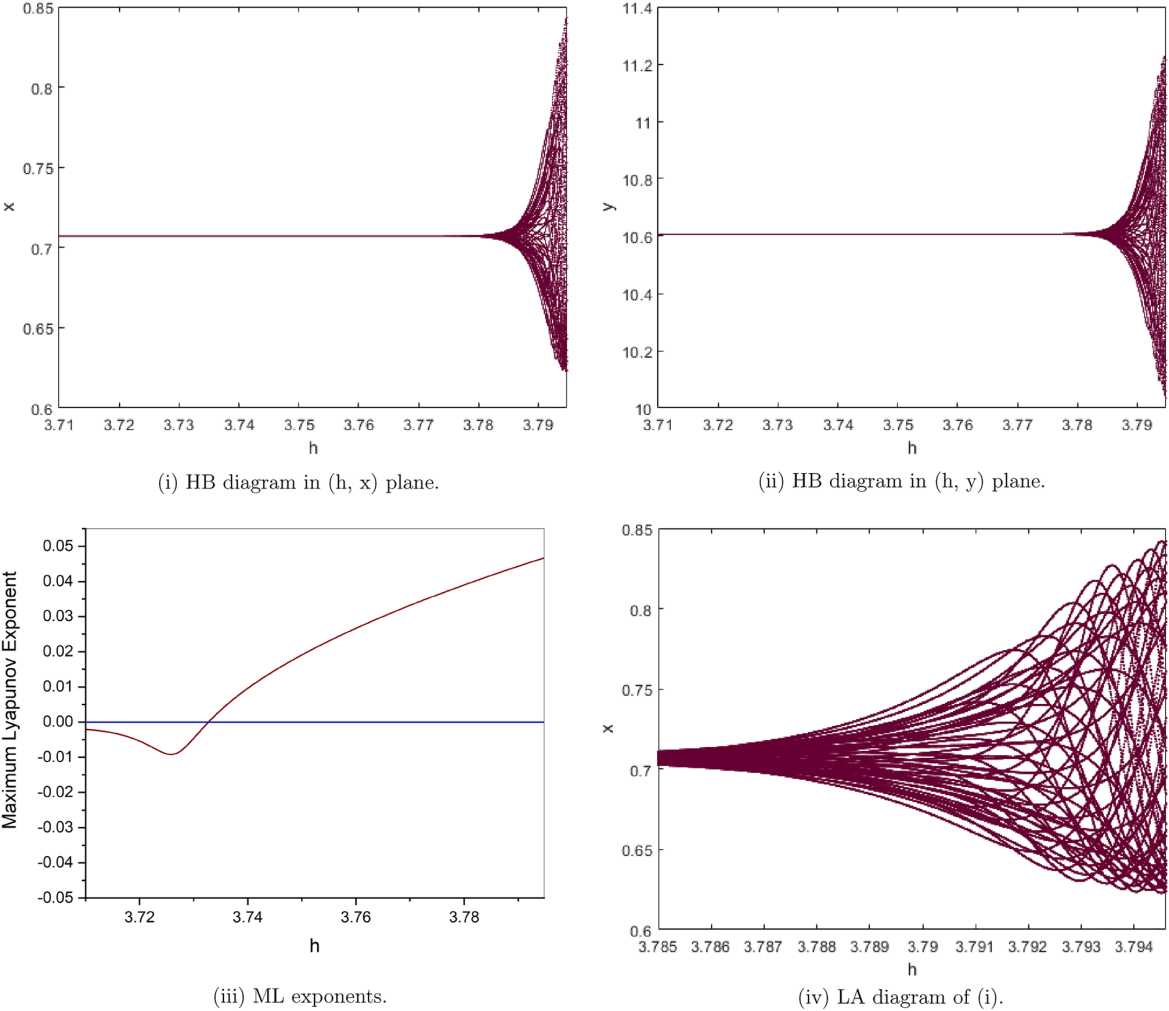
Bifurcation diagrams and ML exponents for the model (i) for values of a = 1.5, b = 0.5, c = 0.1, e = 2, h ϵ [3.71, 3.7948] and LA corresponding to (i) for h ϵ [3.785, 3.7946].

**Figure 9:**
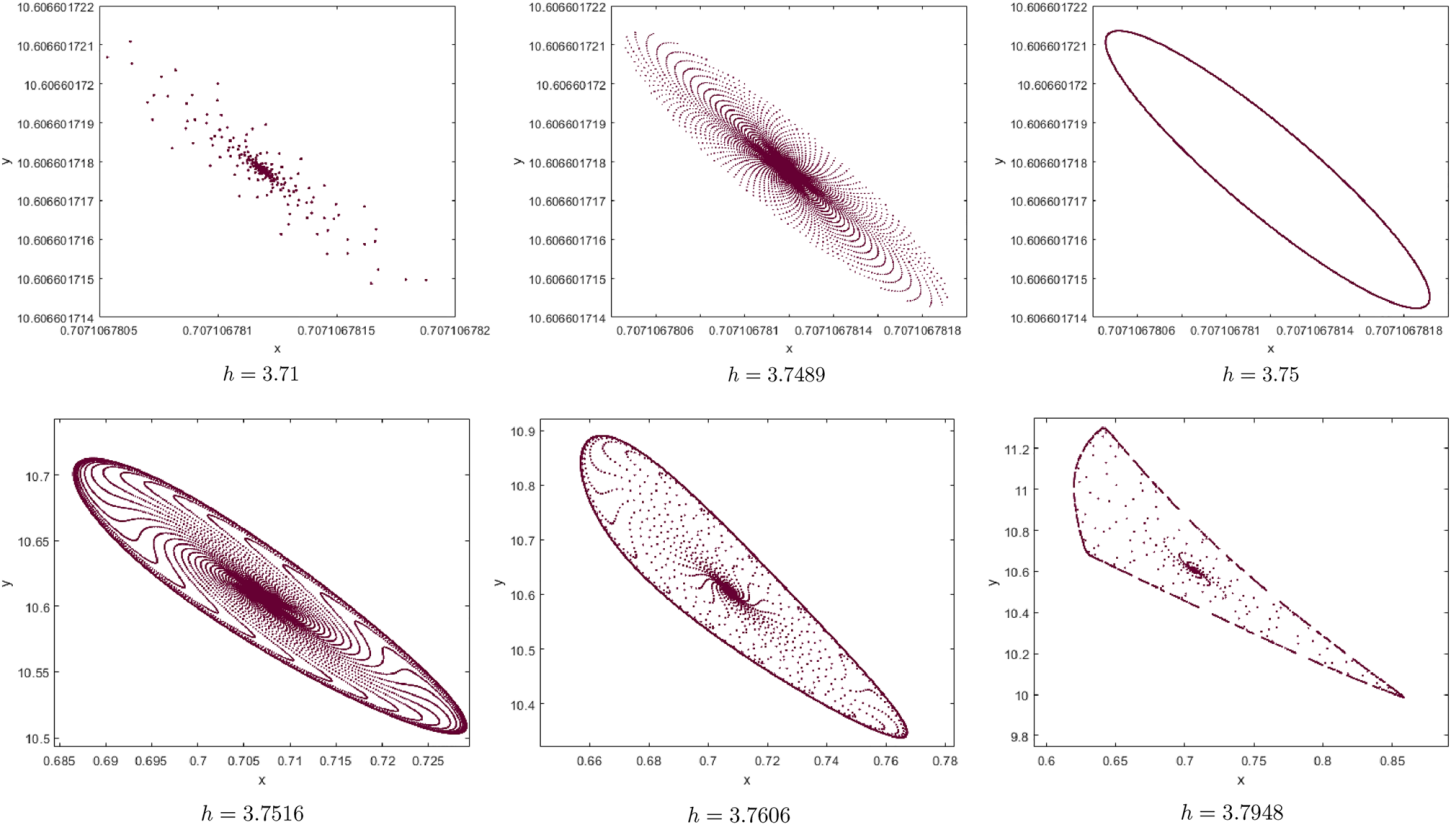
The phase portraits associated with Fig. 8(i), (ii).

*Case 4* We consider h as a parameter and consider the following subcases:

(*I*) $a=1.5, b=0.5, c=0.1, e=2$. By computation the Neimark-Sacker bifurcation with positive fixed point of model (3) shows from the fixed point $p_{1}(x^{*}, y^{*})=(0.7071067810, 10.60660172)$ at $h=3.7328$ with $\Theta =-0.3951153931$ and $(a,b,c,e,h)\in H_{p_{1}}$. This proves that Theorem 4 is correct. From Fig. 8(i), (ii) we observe that the fixed point $p_{1}(x^{*}, y^{*})$ is stable for $3.71\le h<3.7328$ and loses its stability at the Neimark-Sacker bifurcation parameter value $h=3.7328$. Then an attracting invariant cycle bifurcates from the fixed point since $\Theta =-0.3951153931<0$ by Theorem 4. Therefore if $h=3.7328>0$ then the model (3) undergoes a supercritical Neimark-Sacker bifurcation see Table 1. The maximum Lyapunov exponents corresponding to Fig. 8(i), (ii) are calculated and shown in Fig. 8(iii). Figure 8(iv) is a local amplification for $h\in [3.785, 3.7946]$. The phase portraits associated with Fig. 8(i), (ii) are displayed in Fig. 9.

**Table 1:** Numerical values of $\Theta$ for $h>3.7328$.

Value of bifurcation parameter when $h>3.7328$	Numerical value of $\Theta$
3.7489	$$-$$$$0.3909924957<0$$
3.75	$$-$$$$0.3906958052<0$$
3.7516	$$-$$$$0.3902607865<0$$
3.7606	$$-$$$$0.3877366479<0$$
3.7948	$$-$$$$0.3769148619<0$$

(*II*) $a=0.5, b=1.5, c=0.5, e=1.2$. By computation the Neimark-Sacker bifurcation of model (3) shows from the fixed point $p_{1}(x^{*}, y^{*})=(0.7745966692, 0.774596669)$ at $h=0.44$ with $\Theta =-0.0008133888886$, and $(a,b,c,e,h)\in H_{P_{1}}$, which illustrates Theorem 4. From Fig. 10(i), (ii) we observe that the fixed point $p_{1}(x^{*}, y^{*})$ is stable for $0<h<0.44$ and loses its stability at the Neimark-Sacker bifurcation parameter value $h=0.44$, and for $h\in [0.56, 0.58]$ its local amplification is depicted in Fig. 10(iii). The phase portraits associated with Fig. 10(i), (ii) are displayed in Fig. 11.

**Figure 10:**
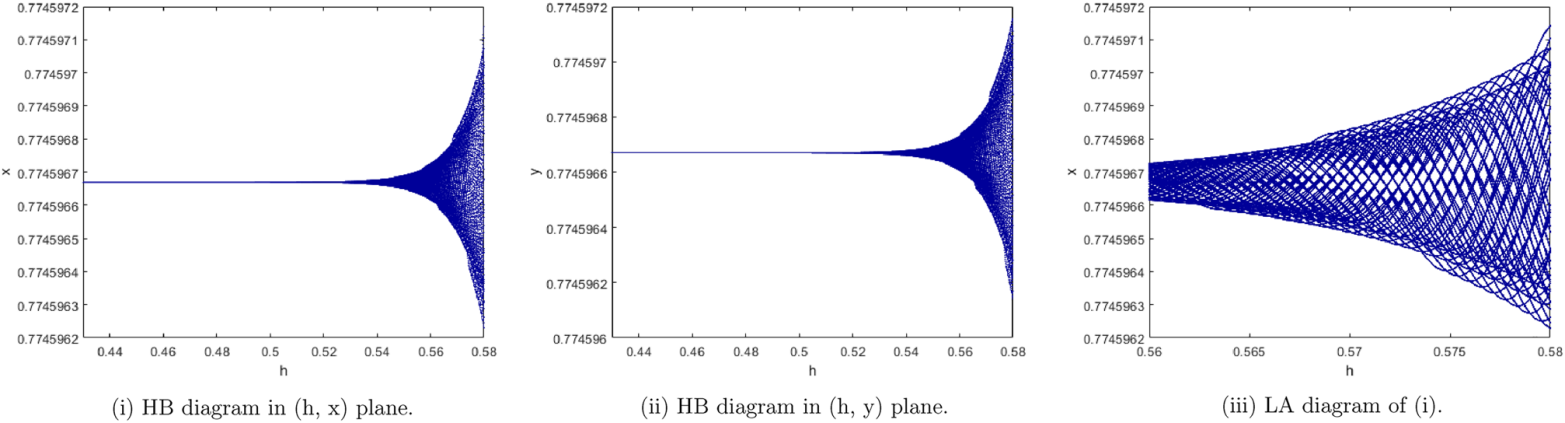
Bifurcation diagrams for the model (i) for values of a = 0.5, b = 1.5, c = 0.5, e = 1.2, h ϵ [0.43, 0.58] and LA corresponding to (i) for h ϵ [0.56, 0.58].

**Figure 11:**
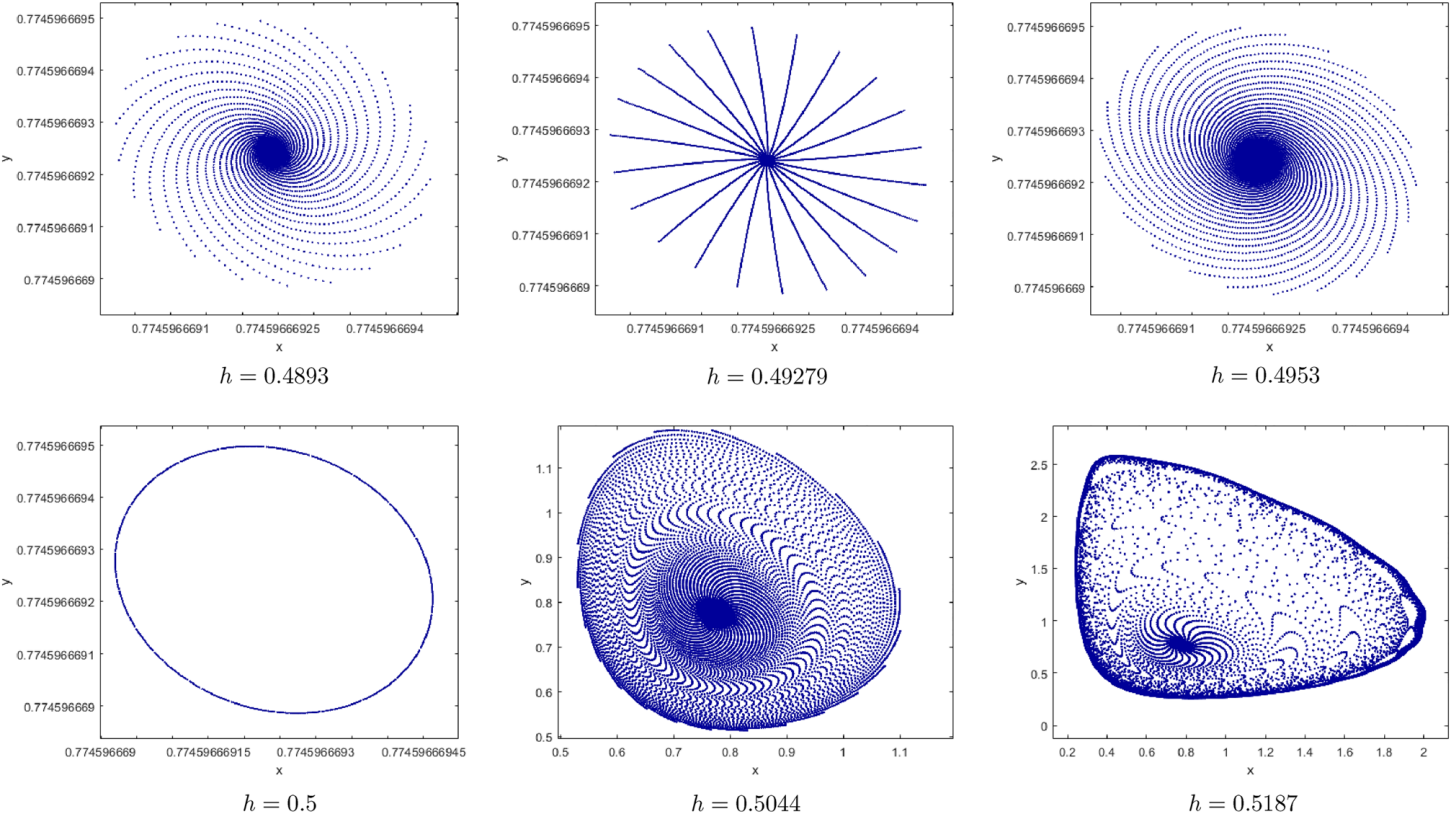
The phase portraits associated with Fig. 10(i), (ii).

**Figure 12:**
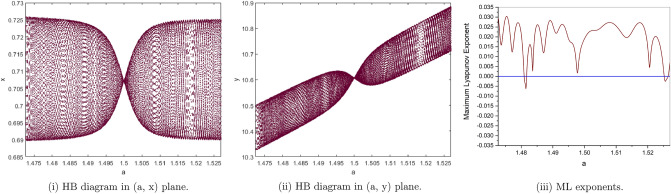
Bifurcation diagrams and ML exponents for the model (i) for values of b = 0.5, c = 0.1, e = 2, h = 3.75, a ϵ [1.4727, 1.527].

*Case 5* We consider a as a parameter and consider the following subcases:

$(I)^{'}$ $b=0.5, c=0.1, e=2, h=3.75$. We get New bifurcation diagrams are obtained, as shown in Fig. 12. This explains that the prey-predator of model (3) experiences a Bidirectional Neimark-Sacker bifurcations in the range $1.4727\le a<1.527$. The system first undergoes chaotic dynamics for small value of *a*. Yet, with increasing value, the chaotic dynamics of the prey-predator system suddenly disappear through the bifurcation of the Neimark-Sacker to a steady state for $a\in \left[ 1.48092, 1.48175\right]$. Next, we find that the dynamics of the predator-prey system jump to a chaotic state through the second Neimark-Sacker bifurcation until it reaches a steady state for $a\in \left[ 1.52525,1.52646\right]$. The maximum Lyapunov exponents corresponding to Fig. 12(i), (ii) are calculated and shown in Fig. 12(iii). Which confirms the dynamic transition in the System (3) from the state of chaos to the stable state and then back again to the state of chaos.

**Figure 13:**
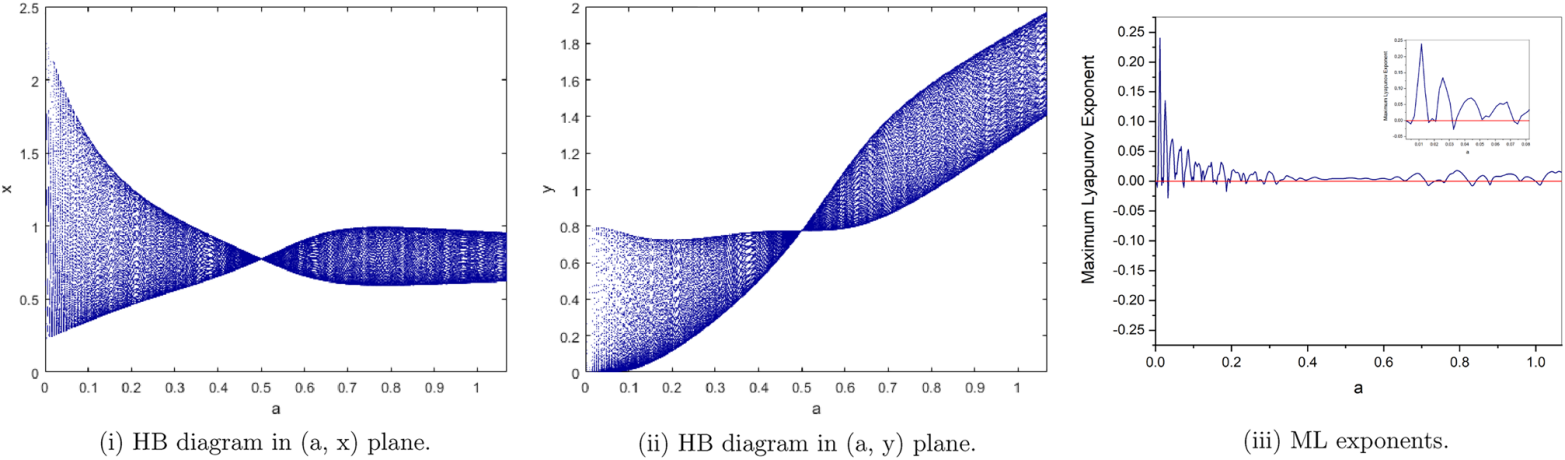
Bifurcation diagrams and ML exponents for the model (i) for values of b = 1.5, c = 0.5, e = 1.2, h = 0.5, a ϵ [0, 1.068].

**Figure 14:**
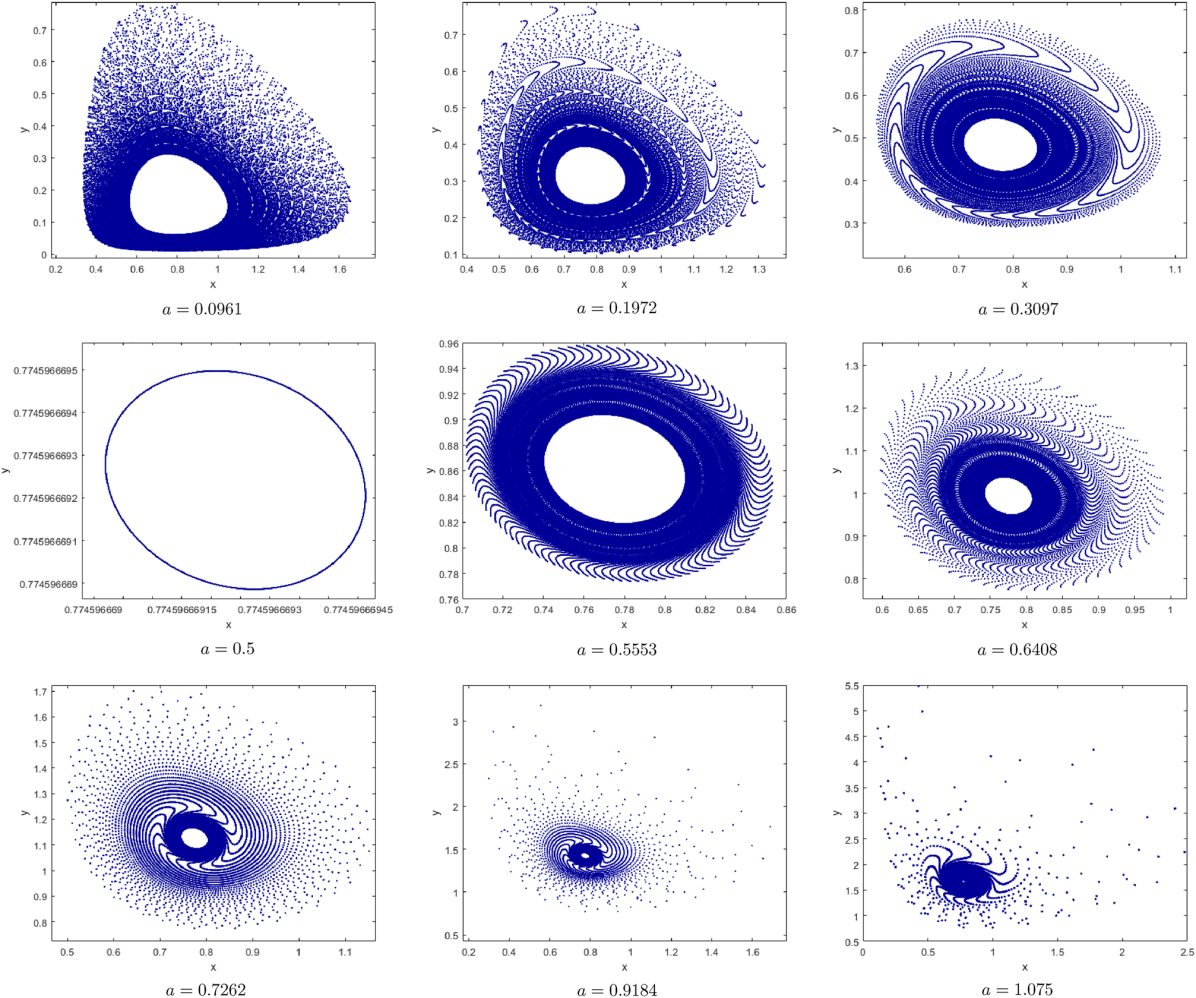
The phase portraits associated with Fig. 13(i), (ii).

$(II)^{'}$ $b=1.5, c=0.5, e=1.2, h=0.5$, from Fig. 13(i), (ii) we observe that the fixed point $p_{1}(x^{*}, y^{*})$ is stable for $0<a<0.00627$. and loses its stability at the Neimark-Sacker bifurcation parameter value $a=0.00627$. The maximum Lyapunov exponents corresponding to Fig. 13(i), (ii) are shown in Fig. 13(iii). The phase portraits associated with Fig. 13(i), (ii) are displayed in Fig. 14.

*Case 6* We consider c as a parameter and consider the following subcases:

$(I)^{''}$ $a=1.5, b=0.5, e=2, h=3.75$. with initial conditions $x^{*}=0.7071067810$ and $y^{*}=10.60660172$. Then, System (3) undergoes both flip bifurcation and Neimark-Sacker bifurcation as c varies in small neighborhoods $c_{1}\simeq 0.09120395559$ and $c_{2}\simeq 0.09942$, respectively. If $a=1.5, b=0.5, e=2, h=3.75$ and $c_{1}=0.09120395559$ the positive fixed point (0.6679877411, 10.98616398) of System (3) and The characteristic equation for (3) is calculated as follows:

$$\begin{aligned} R^{2}+1.57291R+0.572910999=0 \end{aligned}$$

Furthermore, the roots of the above equation are $R_{1}=-1$ and $R_{2}=-0.5729109977$ with $\Gamma _{1}=-0.5333333321$, $\Gamma _{2}=-1.339568037<0$ and $(a,b,c,e,h)\in F_{P_{1}}$. This proves that Theorem (3). Similarly, If $a=1.5,b=0.5,e=2,h=3.75$ and $c_{2}=0.09942$ the positive fixed point (0.7045425810, 10.62979151) of System (3) and The characteristic equation for (3) is calculated as follows:

$$\begin{aligned} R^{2}+1.38805R+0.972240366=0 \end{aligned}$$

**Figure 15:**
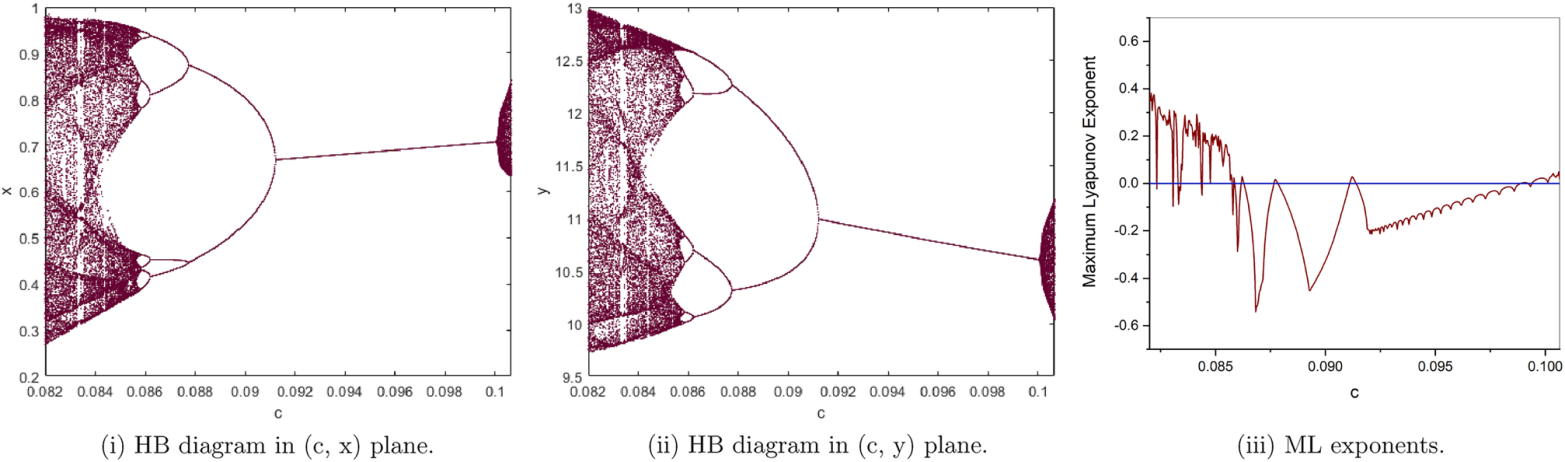
Bifurcation diagrams and ML exponents for the model (i) for values of a = 1.5, b = 0.5, e = 2, h = 3.75, c ϵ [0.082, 0.10065].

Furthermore, the roots of the above equation are $R_{1,2}=-0.6940250000\pm 0.7004067856i$ with $\Theta =-0.4090765553$ and $(a,b,c,e,h)\in H_{p_{1}}$. This proves that Theorem (4). Figure 15 shows bifurcation diagrams and maximal Lyapunov exponents.

**Figure 16:**
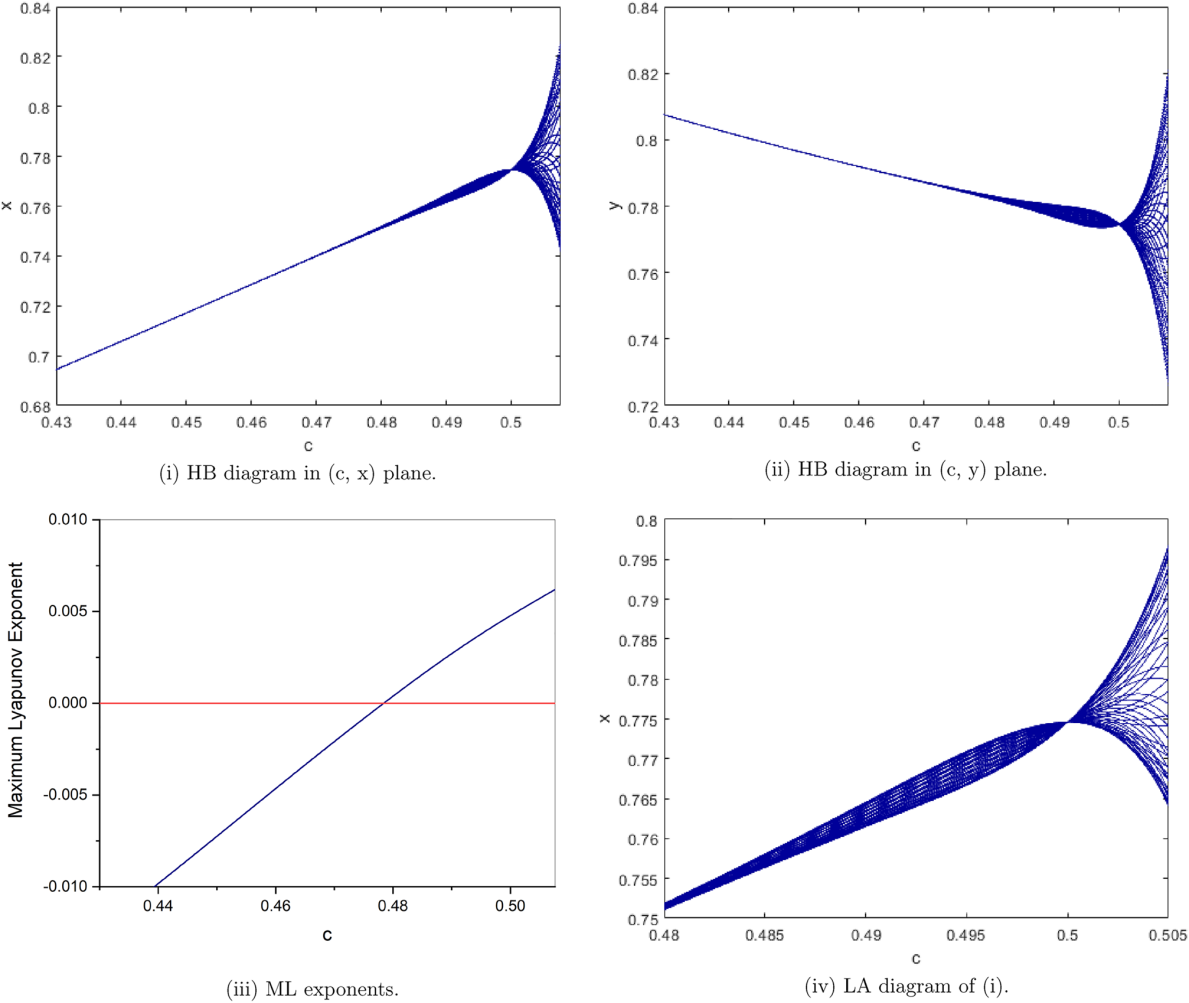
Bifurcation diagrams and ML exponents for the model (i) for values of a = 0.5, b = 1.5, e = 1.2, h = 0.5, c ϵ [0.43, 0.5076] and LA corresponding to (i) for c ϵ [0.48, 0.505].

$(II)^{''}$ $a=0.5, b=1.5, e=1.2, h=0.5$, from Fig. 16(i), (ii) we observe that the fixed point $p_{1}(x^{*}, y^{*})$ is stable for $0.43<c<0.5076$. and loses its stability at the Neimark-Sacker bifurcation parameter value $c=0.47819$. The maximum Lyapunov exponents corresponding to Fig. 16(i), (ii) are shown in Fig. 16(iii), and for $c\in [0.48, 0.505]$ its local amplification is depicted in Fig. 16(iv).

### Chaos control

In order to discuss chaos control for System (3), we apply OGY method and for this taking parameters $a=9,b=2,c=0.25,e=0.5$ and $h=0.5$.

#### Example 3

then System (3) has a unique positive equilibrium point $p_{1}(x^{*}, y^{*})=$ $(0.2672612419, 9.621404709)$ which is unstable. We can take $c_{0}=0.25$ as the nominal value. Then, corresponding controlled system is given by:

28 $$\begin{aligned} x_{n+1}= & {} (1+ah)x_{n}-\frac{hbx_{n}^{2}y_{n}}{e+x_{n}^{2}}, \nonumber \\ y_{n+1}= & {} (1-\left( c-\rho _{1}(x_{n}-x^{*})-\rho _{2}(y_{n}-y^{*})\right) h)y_{n}+\frac{hbx_{n}^{2}y_{n}}{e+x_{n}^{2}}, \end{aligned}$$

where K=$$\left[ \begin{array}{cc} \rho _{1}&\rho _{2} \end{array} \right]$$be gain matrix and $p_{1}(x^{*}, y^{*})=(0.2672612419, 9.621404709)$ is unstable equilibrium point of System (3 ). Furthermore, we have

$$\begin{gathered} J = \left[ {\begin{array}{*{20}c} { - 2.375} & { - 0.125} \\ {7.875} & 1 \\ \end{array} } \right], \hfill \\ C = \left[ {\begin{array}{*{20}c} 0 \\ { - 4.810702355} \\ \end{array} } \right], \hfill \\ \end{gathered}$$

and

$$\begin{aligned} \check{T}=\left[ C:JC\right] =\left[ \begin{array}{ccc} 0 &{} &{} -0.6013377944 \\ &{} &{} \\ -4.810702355 &{} &{} 4.810702355 \end{array} \right] . \end{aligned}$$

Then, it is easy to check that rank of $\check{T}$ is 2, therefore System (28) is controllable. Moreover, the Jacobian matrix $J-LK$ of the controlled System (28) is given by

29 $$J - LK = \left[ {\begin{array}{*{20}l} { - 2.375} \hfill & { - 0.125} \hfill \\ {7.875 + 4.810702355\rho _{1} } \hfill & { - 1 + 4.810702355\rho _{2} } \hfill \\ \end{array} } \right]$$

Then, characteristic equation of (29) is given by

30 $$\begin{aligned} \varvec{\rho }(R)= & {} R^{2}+(1.375-4.810702355\rho _{2})R-1.390625 \nonumber \\&-11.42541809\rho _{2}+0.6013377944\rho _{1}. \end{aligned}$$

Then, the roots of (30) lie inside a unit disk $\left| \mu \right| <1$ if the following conditions are satisfied:

$$\begin{aligned} 0.05263157893\rho _{1}< & {} 0.2092374195+\rho _{2}, \\ 17.30516545> & {} \rho _{1}\ge 6.080193252,0.06062870768 \\ +0.03703703706\rho _{1}> & {} \rho _{2}, \end{aligned}$$

or

$$\begin{aligned} 6.080193252>\rho _{1}>1.507056450,0.09090909096\rho _{1}<\rho _{2}+0.2669237909. \end{aligned}$$

In this case, the lines of marginal stability are given by

$$\begin{aligned}{}&H_{1}:0.6013377944\rho _{1}=1.765625+6.614715735\rho _{2},\\&H_{2}:11.42541809\rho _{2}+2.390625=0.6013377944\rho _{1}, \end{aligned}$$

and

$$\begin{aligned} H_{3}:16.23612044\rho _{2}=0.6013377944\rho _{1}+0.984375. \end{aligned}$$

**Figure 17:**
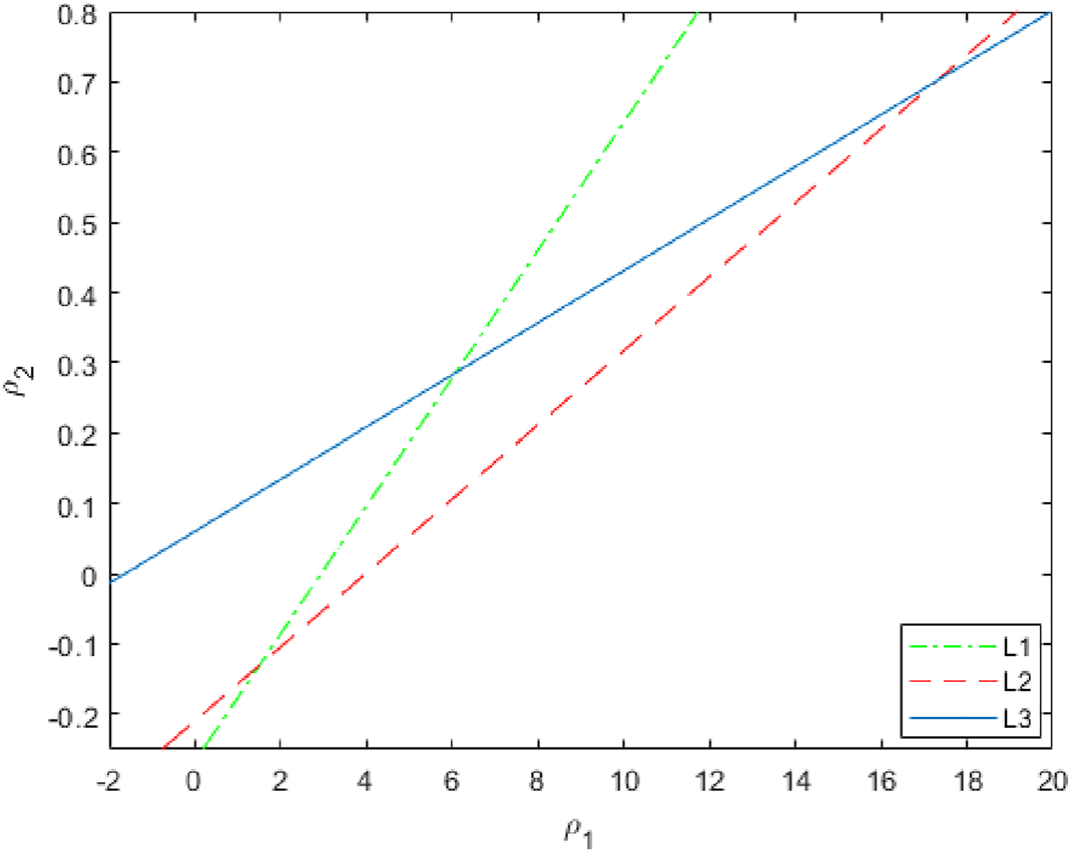
Stability region of the controlled System (28).

Then, the stable triangular region bounded by the marginal lines $H_{1}$, $H_{2}$ and $H_{3}$ for the controlled System (28) is shown in Fig. 17.

**Figure 18:**
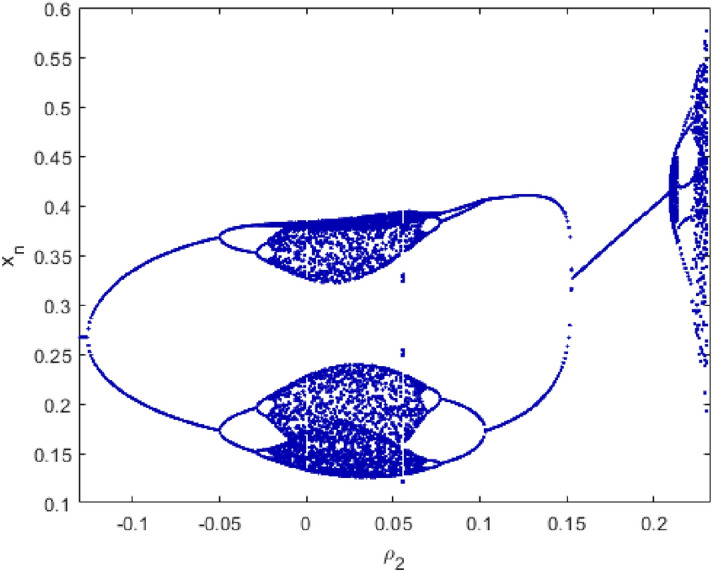
Bifurcation diagrams for the controlled system (28) with $\rho _{1}=1.55$, $p_{1}(x^{*},y^{*})=(0.2672612419, 9.621404709)$ and $\rho _{2}\in [-0.16, 0.3]$.

Next, we take $\rho _{1}=1.55$, then the unique positive equilibrium point of the controlled System (28) is locally asymptotically stable if and only if $-0.1260146999<\rho _{2}<0.1180361151$. Choosing $\rho _{1}=1.55$ and $\rho _{2}\in [-0.16, 0.3]$, then the bifurcation diagrams of the controlled System (28) are shown in Fig. 18.

**Figure 19:**
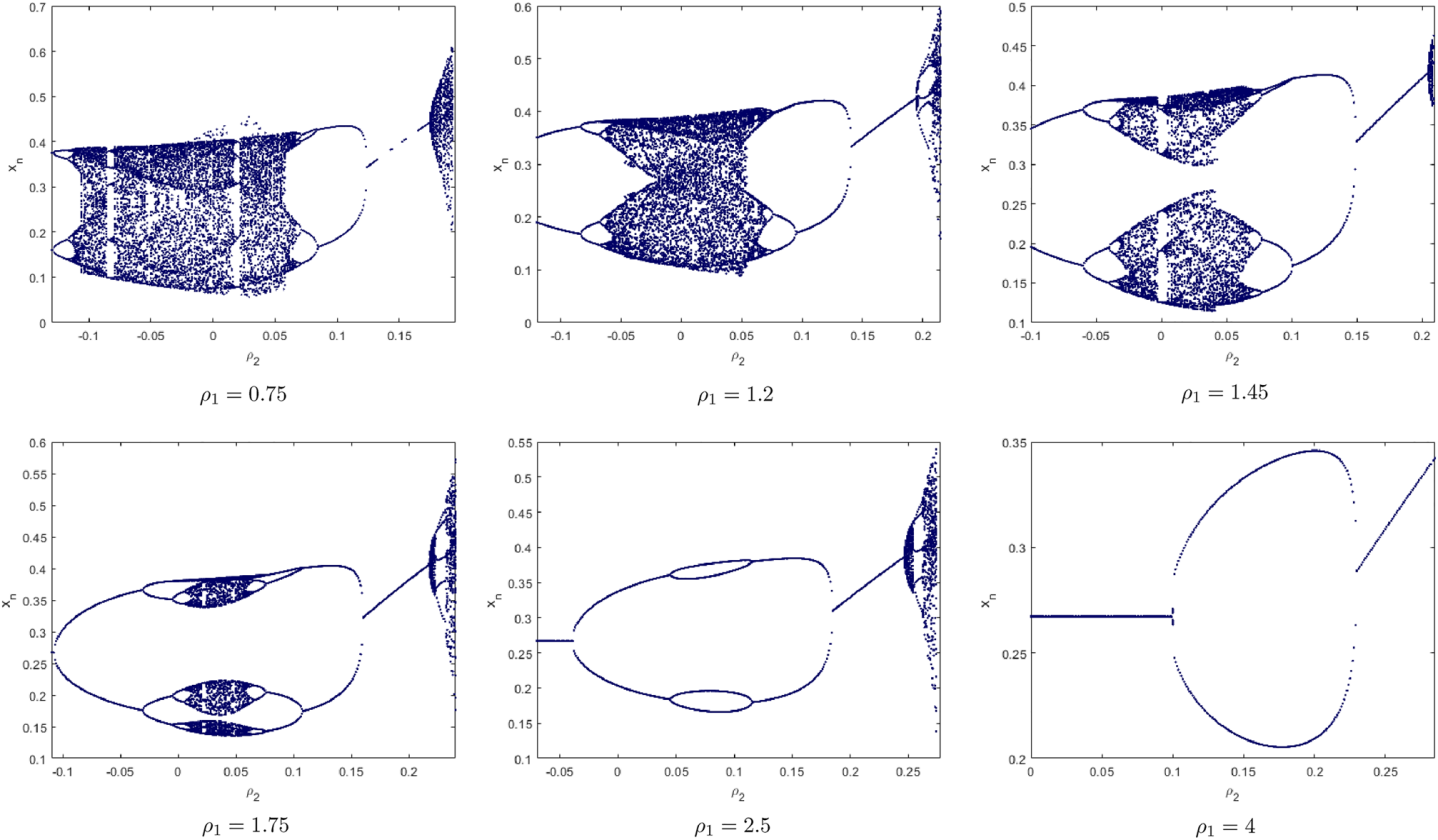
The Phase Chaos of the controlled system (28).

Finally, we will introduce a new concept the phase of Chaos Control bifurcation . We consider $\rho _{2}$ as variable and show the behavior of $x_{n}$ for $\rho _{2}\in [-0.12, 0.3]$. We will choose some values of $\rho _{1}$ as shown in Fig. 19.

## Data Availability

All data used in this manuscript have been presented within the article.

## Code availability

The codes used and/or analysed during the current study available from the corresponding author on reasonable request.
